# Footprints of parasitism in the genome of the parasitic flowering plant *Cuscuta campestris*

**DOI:** 10.1038/s41467-018-04344-z

**Published:** 2018-06-28

**Authors:** Alexander Vogel, Rainer Schwacke, Alisandra K. Denton, Björn Usadel, Julien Hollmann, Karsten Fischer, Anthony Bolger, Maximilian H.-W. Schmidt, Marie E. Bolger, Heidrun Gundlach, Klaus F. X. Mayer, Hanna Weiss-Schneeweiss, Eva M. Temsch, Kirsten Krause

**Affiliations:** 1 Institute for Botany and Molecular Genetics, BioEconomy Science Center, Worringer Weg 3, RWTH Aachen University, Aachen, 52074 Germany; 2 Institute for Bio- and Geosciences (IBG-2: Plant Sciences), Forschungszentrum Jülich, Wilhelm Johnen Straße, Jülich, 52428 Germany; 3 Department of Arctic and Marine Biology, UiT The Arctic University of Norway, Biologibygget, Framstredet 39, Tromsø, 9037 Norway; 4 Helmholtz Zentrum München (HMGU), Plant Genome and Systems Biology (PGSB), Ingolstädter Landstraße 1, Neuherberg, 85764 Germany; 5 Technical University Munich, School of Life Sciences Weihenstephan, Alte Akademie 8, Freising, 85354 Germany; 6 Department of Botany and Biodiversity Research, Faculty Center Biodiversity, University of Vienna, Rennweg 14, Vienna, 1030 Austria; 7 Present Address: Institute of Plant Biochemistry, Heinrich Heine University Düsseldorf, Universitätsstraße 1, Düsseldorf, 40225 Germany

## Abstract

A parasitic lifestyle, where plants procure some or all of their nutrients from other living plants, has evolved independently in many dicotyledonous plant families and is a major threat for agriculture globally. Nevertheless, no genome sequence of a parasitic plant has been reported to date. Here we describe the genome sequence of the parasitic field dodder, *Cuscuta campestris*. The genome contains signatures of a fairly recent whole-genome duplication and lacks genes for pathways superfluous to a parasitic lifestyle. Specifically, genes needed for high photosynthetic activity are lost, explaining the low photosynthesis rates displayed by the parasite. Moreover, several genes involved in nutrient uptake processes from the soil are lost. On the other hand, evidence for horizontal gene transfer by way of genomic DNA integration from the parasite’s hosts is found. We conclude that the parasitic lifestyle has left characteristic footprints in the *C. campestris* genome.

## Abstract (web summary)

Parasitic lifestyles leave unique genomic footprints. Here, the authors describe the genome sequence of a parasitic plant, *Cuscuta campestris*, and find that gene losses and host gene acquisitions reflect the independence from photosynthesis and the ability to retain and express chunks of foreign genomic DNA.

## Introduction

**Fig. 1:**
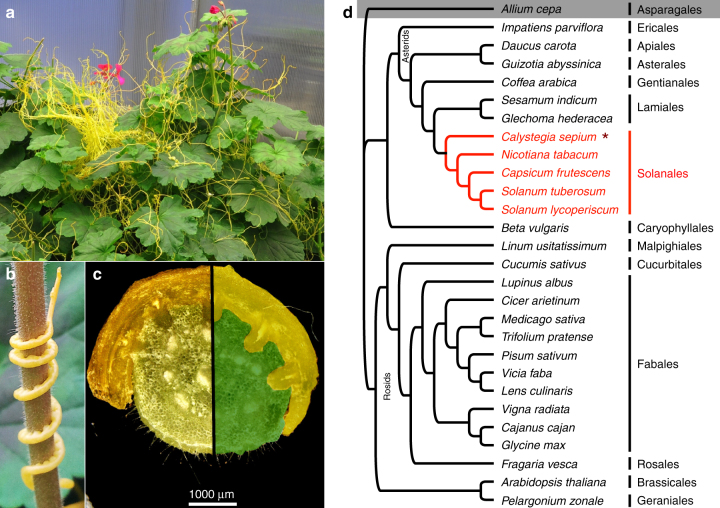
*Cuscuta campestris*—host plant relationship. **a** *Cuscuta campestris* (yellow) infecting *Pelargonium zonale*. **b** Close-up of a parasitic vine entwining and infecting a host shoot. **c** Cross section through an infection site. The right half of the micrograph was overlayed with color to emphasize the border between *C. campestris* (yellow) and the *P*. *zonale* stem (green). **d** Simplified phylogenetic tree of reported host plant species of *C. campestris*, showing the variety of angiosperm orders (named on the right) that contain host species (according to refs. ^1–3^). The gray background highlights the single described monocot host. The asterisk indicates the closest phylogenetic relative of *C. campestris* (Convolvulaceae) within the host groups

The field dodder (*Cuscuta campestris* Yunck.) belongs to the genus *Cuscuta* (Convolvulaceae, Solanales) that comprises about 200 parasitic plant species, many of which are devastating agronomic weeds^1^. As a result of their parasitic nature, which consists of winding around and infecting the aerial parts of their host plants, they have a severely altered morphology: they do not possess roots or proper leaves (Fig. 1a, b), but instead have specialized infection organs for host attachment and intrusion called haustoria. The haustoria grow into the host tissue and connect the vascular systems of the two plants (Fig. 1c), thus enabling the parasite to absorb water, photoassimilates, and other organic and inorganic compounds from its host. In contrast to most other parasitic plants and to plant pathogens in general, dodder species, including *C. campestris*, have a remarkably broad host range, which includes annuals, shrubs, and trees representing different lineages across the plant kingdom (Fig. 1d)^1–3^.

For several *Cuscuta* species, reductions in the size and coding potential of the plastid genomes have been reported^4, 5^. Unfortunately, aside from several recent transcriptome analyses^6–9^ there is very little information on the other cellular genomes of these shoot parasites. To an even larger extent, the phenomenon of plastid genome downsizing is also visible in root-parasitizing Orobanchaceae that cannot support themselves by photosynthesis^10^. In contrast, in the latter group some nuclear genomes appear to have increased in size and potentially in function^10^. Still, a full nuclear genome sequence has not been published for any parasitic plant to date.

To close that knowledge gap, we describe here the genome of *C. campestris* as derived from shotgun sequencing, genome assembly, gene annotation, and repetitive element analysis. This genome bears footprints of *Cuscuta’s* parasitic lifestyle in the form of horizontally acquired genetic material from the host and in the form of gene losses that are in agreement with the differences in their physiology, compared to non-parasitic plants.

## Results

### Karyology and genome characteristics

**Fig. 2:**
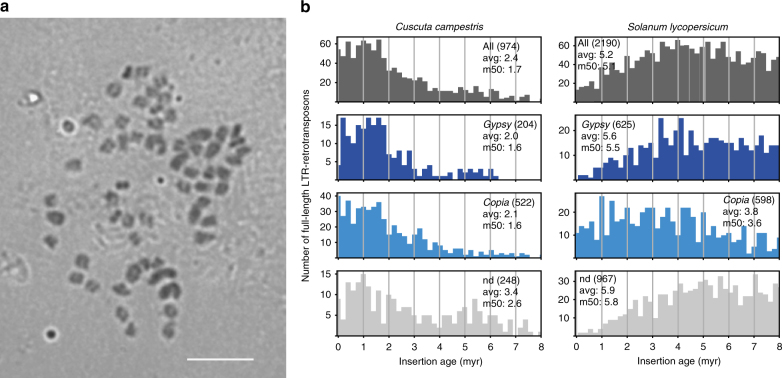
Genome features. **a** Feulgen-stained metaphase chromosomes of *C. campestris* (2*n* = 56). The scale bar represents 10 µm. **b** Insertion age distribution of *C. campestris* full-length LTR retrotransposons in comparison to tomato (*Solanum lycopersicum*) as another Solanales representative. Insertion events of long terminal repeat (LTR) retrotransposons were dated by comparing the divergence between the 5′ and 3′ long terminal repeats^61^. A random mutation rate of 7*10^−9^ was used to convert the sequence divergence into age^62^ (in million years)

*C. campestris* possesses 2*n* = 56 small chromosomes as revealed by Feulgen staining and has a 1C-value of 581 megabase pairs (Mbp; or 0.594 when given in pg) based on our flow cytometric measurements. Chromosome sizes range from 0.8 to 1.5 µm, with only a few (10–12) larger chromosomes (Fig. 2a). Whole-genome shotgun sequencing was performed using Illumina paired-end data coupled with Illumina mate pair libraries of different insert sizes and PacBio long reads for scaffolding. This provided a size of just under 477 Mbp for the filtered and assembled draft genome sequence, representing about 86% of the k-mer-based genome size estimate of 556 Mbp and 82% of the 581 Mbp size indicated by flow cytometry. It contains 6907 scaffolds with sizes up to 5,342,339 bp (Supplementary Table 1), featuring an N_50_ (minimum scaffold length at 50% of the genome) of 1.38 Mbp. The genome contains 44,303 predicted high confidence gene loci, excluding splice variants, that were supported either by transcriptome data from this parasite and/or sequence similarity to known sequences. A BUSCO analysis to assess gene completeness^11^ indicated, that of 1440 genes typically conserved in plants, 82.1% were present in the assembly, and that 59% of these 1440 genes were present in a duplicated form (Supplementary Table 2).

Of the assembled genome sequence, 46.2% (221 Mbp) was identified as transposon derived with a strong dominance of long terminal repeat (LTR) retrotransposons (96% of all mobile genetic elements). The amount and composition of the repetitive genome fraction (Supplementary Table 3) is similar to what was found in solanaceous plants^12^ and lends support to a good assembly, capturing the repetitive content that is expected for the genome size. Interestingly, the insertion age distribution of all full-length LTR retrotransposons and their superfamily subgroups shows in comparison to other Solanales, like tomato, a more recent amplification wave around 1.5 million years ago (mya) with only minor differences between their subgroups (Fig. 2b) which could have been triggered by a polyploidisation event^13^.

### Whole-genome duplication

**Fig. 3:**
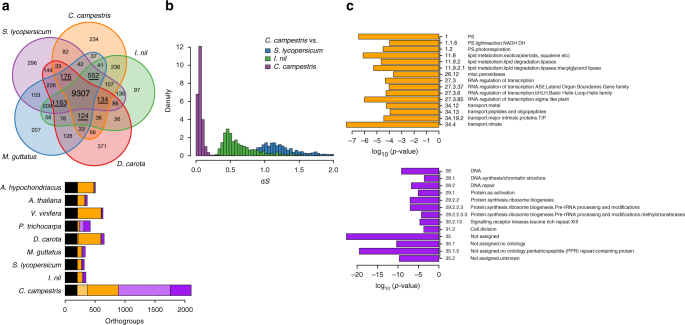
Duplication and loss of genes in the *C. campestris* genome. **a** Overview over 14,626 asterid orthogroups from *C. campestris*, *Ipomoea nil*, *Solanum lycopersicum*, *Mimulus guttatus,* and *Daucus carota*. The Venn diagram shows the number of overlapping and exclusive orthogroups found in the five species. The bar diagram below summarizes gene content in orthogroups in *C. campestris* and eight divergent dicot species. Indicated in black are orthogroups with exactly one member in each of these species (202 OGs). Orthogroups missing in the respective species only, but that are otherwise present in all species once (exactly 0:1, named:other species, striped) or more than once (ratio 0:1 or more, solid color) are shown in orange and are underlined in the Venn diagram. Duplicated orthogroups are defined as having exactly 2:1 genes per species (striped purple) or as having a ratio of at least 2n:n with at least one gene in every species (solid purple). All above ratios are (named species):(other species). **b** Synonymous substitution rate (d*S*) analysis. Density of d*S* rates for *C. campestris* paralogs are shown in purple, and for 1:1 orthologs with *I. nil* and *S. lycopersicum* in green and blue, respectively. **c** Functional enrichment analysis of lost and duplicated genes. MapMan categories for genes that are significantly enriched are depicted using the logarithmic values of their *p* values. The orange bars represent lost genes that are present in one or more copies in the eight other dicot species shown in **a** but are lost in *C. campestris* (ratio 0:1 or more). Similarly, purple bars represent categories significantly enriched for duplicated genes (at least 2*n*:*n*)

An orthogroup analysis was performed between *C. campestris*, the related species *Ipomoea nil* and ten other species including Rosids, Asterids, and more distant Monocots (Supplementary Data 1). This revealed that *C. campestris* indeed has duplicate copies of many genes when compared to other species, including, but not limited to, representatives of the Solanales (Fig. 3a). The peak synonymous substitution rate (d*S*) was on average much lower between paralogs in *C. campestris* (0.05), than between genes of the parasite and their orthologs in *I. nil*^14^ (0.5, split about 55 mya^15^) and *Solanum lycopersicum* (1.2, split > 80 mya^15, 16^) (Fig. 3b). The majority of genes in *C. campestris* (59% compared to 23% in *Arabidopsis thaliana*) had a syntenic paralog, and paralogs with a d*S* value around the peak at 0.05 were especially likely to be syntenic (Supplementary Fig. 1). Further, the most common ratio between genes in syntenic blocks was two homologs in *C. campestris* to one in *S. lycopersicum* (Supplementary Fig. 2). Functional enrichment analysis in orthogroups with twice or more the genes in *C. campestris* than in other dicot species showed, furthermore, that tendencies towards conserving duplicated genes are particularly conspicuous in MapMan categories including “DNA” and “protein” functions. Taken together, the high proportion of gene duplicates featuring a low d*S* value and the recent LTR-retrotransposon proliferation (Fig. 2b) point to a recent genome duplication event.

### Gene loss

To uncover further manifestations of the parasitic lifestyle in the genome in the form of gene or pathway losses, 1027 gene families that lack an ortholog in *C. campestris* but are otherwise conserved in all other tested dicots (Fig. 3a) were subjected to a functional enrichment analysis on the basis of MapMan categories^17^. Functional enrichments were particularly observed for the categories “photosynthesis”, “RNA”, “stress”, “transport”, and “lipid metabolism” (Fig. 3c), which contrasts with the categories enriched for duplications.

**Fig. 4:**
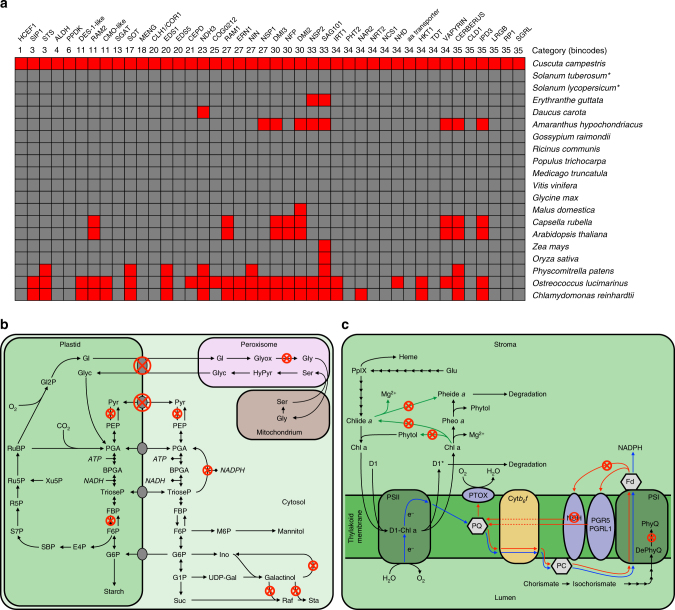
Comparison of gene losses between *C. campestris* and autotrophic plants. **a** Presence (gray) and absence (red) of 42 lost genes connected to photosynthesis, metabolism, transport processes, and symbiotic interactions in 19 sequenced genomes (from Phytozome v.12.0) of the angiosperms, lower plants, and green algae. Species names are shown on the right in the order of increasing evolutionary distance from *C. campestris* (from top to bottom). Gene abbreviations and MapMan bincodes are given on the top. Full gene names and descriptions of the bincodes are given in Supplementary Table 5. Asterisks behind species names indicate that the sequences from these species were used as query for manual homology searches against the *C. campestris* genome. **b** Schematic view of primary carbon metabolism, i.e., Calvin cycle, photorespiration, plastid and cytosolic glycolysis and gluconeogenesis, and synthesis of the compatible solutes mannitol, raffinose and stachyose. Enzymatic reactions for which genes are missing are indicated by circled red crosses. E4P erythrose-4-phosphate, F6P fructose-6-phosphate, FBP fructose-1,6-bisphosphate, G1P glucose-1-phosphate, G6P glucose-6-phosphate, Glyc glycerate, Gly glycine, Gl glycolate, Gl2P glycolate-2-phosphate, Glyox glyoxylate, HyPyr hydroxypyruvate, Ino inositol, M6P mannose-6-phosphate, PEP phosphoenolpyruvate, PGA 3-phosphoglycerate, BPGA 1,3-bisphosphoglycerate, Pyr pyruvate, Raf raffinose, R5P ribose-5-phosphate, Ru5P ribulose-5-phosphate, RuBP ribulose-1,5-bisphosphate, S7P sedulose-7-phosphate, SBP sedulose-1,7-bisphosphate, Ser serine, Sta stachyose, Suc sucrose, TrioseP triose phosphates, UDP-Gal UDP-galactose, Xu5P xylulose-5-phosphate. **c** Schematic view of photosynthetic electron transport, chlorophyll regeneration, and degradation pathways and phylloquinone synthesis. Enzymatic reactions for which genes are missing are indicated by circled red crosses. Chl a chlorophyll a, Chlide *a* Chlorophyllide *a*, Cyt*b*_*6*_*f* cytochrome *b*_*6*_*f* complex, D1 D1 protein of photosystem II, D1* damaged D1 protein, DePhyQ demethylphylloquinone, e^−^ electron, Fd ferredoxin, Glu glutamate, NDH NADH-dehydrogenase complex, PC plastocyanin, PGR5/PGRL1 proton gradient regulation 5/PGR5-like photosynthetic phenotype 1-complex, Pheide *a* pheophorbide *a*, Pheo *a* pheophytin *a*, PhyQ phylloquinone, PpIX Protoporphyrin IX, PQ plastoquinone, PS I photosystem I, PS II photosystem II, PTOX plastid terminal oxidase

In a second, independent approach, 1736 gene losses were found in *C. campestris* relative to *I. nil* when performing one-to-one blast searches with the ~23,700 conserved proteins of this closest available non-parasitic relative of *Cuscuta*. In total, 42 random proteins from a broad cross section of functional categories that were contained in both lists, and that were of particular interest due to their involvement in processes that distinguish *C. campestris* from non-parasitic plants, were manually checked for their conservation across the plant kingdom (Fig. 4a). With the exception of some genes involved in symbiotic interactions that are lacking in Brassicales and Caryophyllales^18^, but are present in all other plant lineages, these genes are conserved across land plants, supporting the assumption that their function has become obsolete due to the parasitic lifestyle. While *C. campestris* has lost genes that are needed for photosynthesis at higher light intensities (Fig. 4b, c), the genes for the photosynthetic light reactions (linear and one of the two cyclic electron transport pathways) and complete chlorophyll and carotenoid synthesis pathways were retained. Similarly, all amino acid and fatty acid synthesis genes, as well as co-enzyme and vitamin pathway genes are present, although the parasite is thought to obtain all these compounds from its hosts^19^.

### Horizontal acquisition of genes

**Fig. 5:**
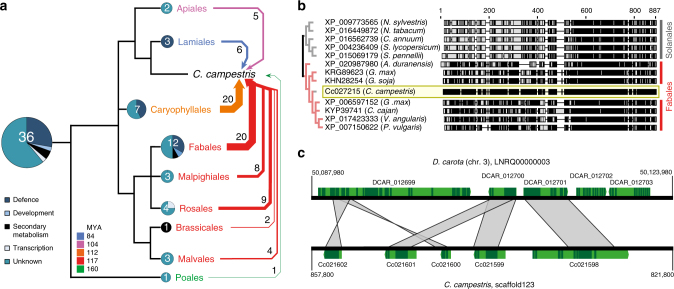
Horizontal gene transfer (HGT) in *C. campestris*. **a** Depiction of the phylogenetic origin of 36 HGT events and their functional category assignment. Phylogenetic orders from which HGT originated are color-coded according to their evolutionary distance to *Cuscuta* using the median values in million years (MYA) for the split as predicted by Timetree (http://timetree.org) and supported by a species tree calculated using Astral-II (Supplementary Fig. 5). White numbers refer to the number of genes per donor order, black numbers to the right indicate the number of genes in the recipient, *C. campestris*. Cake diagrams represent functional assignment of donor genes in MapMan bins^17^. **b** Phylogenetic placement of HGT candidate protein Cc027215, a G-type lectin S-receptor-like serine/threonine-protein kinase, Callus Expression of RBCS 101 (CES101). The amino acid sequence of Cc027215 (highlighted in yellow) is nested deeply within a cluster of orthologous proteins from the hypothetical donor order Fabales (in red), while all orthologs from Solanales species (in gray), that are much more closely related to *C. campestris*, are located on the second branch. The schematic sequence comparison shows identities relative to Cc027215 in black boxes, discrepancies in gray boxes and gaps in the alignment as black lines. Bootstrap values after 1000 replicates were 86% or higher for all nodes. **c** Nucleotide sequence comparison of a 36 kb region of chromosome 3 from *Daucus carota* (top) and a region of *C. campestris* scaffold123. Coding sequences are shown in dark green and introns as well as up- and down-stream untranslated flanking regions are shown in light green. Areas with very high similarity, indicating conserved remnants of a previous horizontal DNA transfer between both species are indicated by gray fields. Within the depicted area, a gene duplication (*Cc021601* and *Cc021599* both mapping with all three exons and two introns to *DCAR_012700*), a larger sequence insertion between *Cc021599* and *Cc021598*, as well as sequence inversions can be seen

It has been shown that root-parasitic Orobanchaceae acquired a number of genes by horizontal gene transfer (HGT) from their hosts^20^. Similarly, two incidents of HGT have been reported on the basis of transcriptome studies in *Cuscuta pentagona* and *Cuscuta australis*. One example is an albumin gene acquired from legumes^21^ and the other a strictosidine synthase-like gene originating from the Brassicaceae^22^. In both cases, the presence of introns in contigs or genomic PCR amplicons suggested that these integrations occurred via DNA uptake. Large-scale transport of phloem-mobile transcripts appears to occur in both directions across the host–parasite border, tentatively suggesting that an integration mechanism via RNA or cDNA is also possible^7, 23^. So far, however, horizontal DNA transfer in *Cuscuta* beyond these cases of individual genes has not been analyzed. In search for horizontally acquired genes in the *C. campestris* genome, we identified, in addition to the above mentioned horizontally transferred genes, 64 novel high confidence HGT candidates from at least 32 different donor sequences that show a functional bias towards defence reactions or have unknown functions in their hosts (Supplementary Table 4). All of them group phylogenetically with orders other than the Solanales (Fig. 5a, b, Supplementary Table 4, Supplementary Figs. 3, 4), where the genus *Cuscuta* is phylogenetically placed (Supplementary Fig. 5). A majority of these events can be traced back to the preferred host orders Fabales and Caryophyllales suggestive of HGT between host and parasite (Fig. 5a, b). To refute that these putative HGT events are a result of gene duplication or other phenomena, the Notung software package was used to infer models that best explain the disagreement between species trees and gene trees^24^. For all HGT candidates, the Notung models confirm acquisition by HGT (Supplementary Figs. 3, 6, 7). This conclusion is further supported by the observation that a small part of the transposable element (TE) complement was identified via non-*Cuscuta* repeat library sequences, often from soybean and *Medicago truncatula*. Intriguingly, two *C. campestris* genes that are adjacent to each other show highest homology to two neighboring genes from the *Daucus carota* (carrot) genome^25^, *Cc021598*, and *Cc021599* (Fig. 5c). In the vicinity of these conserved genes are remnants of further genes that were not fully retained after transfer. Moreover, the two introns in the gene *Cc021599* (Fig. 5c) are located in the same position as in its ortholog from carrot (Supplementary Fig. 8). In addition, this gene appears to have undergone duplication in *C. campestris* or its recent ancestor. The duplicate sequence, *Cc021601* is 85% identical to *Cc021599* (pairwise identity of the gene sequence, CDS pairwise identity is 93.9%) and both of them have a high homology (Fig. 5c) to the same carrot gene (with just under 50% pairwise identity for the CDS).

Reads representing many of the HGT candidates were found in one or more RNAseq samples that were used to support gene calling (Supplementary Data 2), indicating that the transferred genes are expressed. Some of them, moreover, showed strong up- or downregulation in haustorial vs. control tissue (Supplementary Fig. 9), suggesting that their gene products may play a role during the infection process.

## Discussion

Genome sizes and chromosome numbers vary considerably between different *Cuscuta* species (1C = 470 Mbp^26^–32 Gbp^27^), and occasionally different sizes based on flow cytometry have even been reported for the same species (e.g., refs. ^26, 27^ for *C. campestris*). The 1C value of 581 Mbp that we determined for *C. campestris* is within this range at its lower end. *C. campestris* reveals signs of a whole-genome duplication around 1.5 mya (Fig. 3, Supplementary Figs. 1 and 2).

It has often been suggested and experimentally verified for some systems that the tight epigenetic control of transposons is impaired after polyploidisation events^28^. In line with this, the insertion age distribution in *C. campestris*, and—with it—LTR-retrotransposon activity, displays two distinct peaks. The younger more prominent event occurred around 1.5 mya where the majority of LTR insertions originated and that affected all subgroups likewise (Fig. 2b). In addition, the remnants of an older amplification wave around 5–6 mya ago is visible. In contrast, overall LTR-retrotransposon activity in tomato only peaks between 3.5 and 6.5 mya and only some Ty1/*copia* elements seem younger. That the younger peak coincides with a recent genome duplication event was also inferred from substitution rate and synteny analyses as well as a BUSCO analysis (Fig. 3, Supplementary Figs. 1, 2, Supplementary Table 2).

Besides finding 59% duplicated genes in a set of 1440 conserved genes, BUSCO yielded higher counts for missing genes than with the “classical” genomes of related photosynthetic plants (Supplementary Table 2). Further analyses revealed that exhaustive gene losses have occurred in *C. campestris*. The gene classes that were indicated to be lost by the BUSCO analysis coincide with those identified by other means (Figs. 3, 4), explaining, at least in part, the poorer performance of the software in this respect.

Many of these nuclear-encoded losses in the parasite were found to be associated with one of three categories: redundant pathways or redundant functions, high photosynthetic production rates, and the interaction with the biotic and abiotic environment (Figs. 3c, 4). For instance, genes for the NDH-dependent cyclic electron transport pathway^29^ and genes for a dephytylase and a dechelatase needed for chlorophyll turnover^30^ are important to adjust photosynthesis in situations of high fixation rates and fluctuating light conditions (Fig. 4). They are apparently superfluous for *C. campestris*, where photosynthesis rates are so low that the CO_2_ compensation point is never reached^31^. This is in line with the retention of the photosynthesis genes encoded on the plastid genome (with few exceptions)^4, 5^ (see also Supplementary Fig. 10) and imposes the question why these pathways are still complete in the parasite even though they appear to be too inefficient to enable host-independent growth. Developing seeds of autotrophic plants, many of which are green during embryogenesis and show some photosynthetic activity despite depending heavily on imported nutrients, are in an analogous situation. Interestingly, it has been shown that re-fixation of CO_2_ released during fatty acid biosynthesis, mitochondrial respiration and other processes significantly improves carbon efficiency of seeds and their growth rate^32, 33^. Thus, it is likely that the low photosynthesis levels in field dodder (and other dodder species) primarily facilitate re-fixation of produced CO_2_. This reduces carbon loss and improves the chances of the parasite successfully bridging the distance from one host to the next in situations where susceptible hosts are not located in close proximity (as in natural ecosystems) or during germination before the parasite has established its first feeding connection.

Examples of gene losses in *C. campestris* that mediate environmental responses in autotrophic hosts are mostly connected to soil–root interactions and therefore might be connected to the loss of a root system in dodder species. To this end, iron transporters that are responsible for uptake of iron from the soil^34^, as well as proteins involved in the establishment of symbiotic relationships between plants and mycorrhizal fungi, were found to be dispensable in *C. campestris* (Fig. 4a). Genes belonging to the latter group were previously found missing in Brassicaceae and Caryophyllales^18^ but are present in most other families (Fig. 4a). One nitrate uptake system of roots (the high affinity NRT2/NAR2 complex) along with the CEPD peptides regulating its expression^35, 36^ is also missing in *C. campestris* (Fig. 4a). Overall, it appears that typical known traits of the parasite such as its nutritional dependency on the host plant and the ensuing release from the necessity of fixing CO_2_ photosynthetically are clearly reflected by changes and gene losses in the genome. This nourishes the hope that, vice versa, gene losses in other than the functional categories described here may also reveal other, more obscure, consequences of parasitism.

As a result of the close physical connection between parasite and host, the exchange of nucleic acids across species borders is prone to occur. Such events, known as HGT, were already inferred from recent transcriptome analyses for *Cuscuta* and other parasites^20–23, 37^, as well as for grafting partners^38^. Although the identification of true HGT events and the bona fide identification of the gene donor can be challenging, the analysis of the *C. campestris* genome provided unambiguous indications that HGT events have at least in part occurred as a result of an uptake and integration of larger stretches of genomic DNA from hosts that are not closely related to the order Solanales in which *Cuscuta* is nested (Fig. 5, Supplementary Fig. 5). The exchange of DNA between parasitic plants and their hosts is well known from the analysis of mitochondrial DNA and these exchanges were also shown to preferentially involve bigger pieces of DNA rather than gene-by-gene transfers^39^. Alas, examples from the nuclear genome are considerably scarcer. An HGT event in *C. campestris* that stands out from previous accounts in that it encompasses a larger region on the genome, bears signs of conservation and even gene duplication in some parts and post-transfer changes in others (Fig. 5). Whether the conserved and duplicated transferred genes have undergone neo-functionalization to adapt to infection-related tasks or whether their products may help to develop a molecular camouflage, enabling the parasite to remain unnoticed during an attack, remains speculative. The elevated expression of some of them in early developing haustoria provides a first hint at their involvement in the infection process that must be scrutinized further in the future.

In summary, the *C. campestris* genome provides clues—hidden in the type and organization of the coding sequences—on evolutionary, physiological and developmental aspects connected to parasitism in plants. This study will pave the way for genome-enabled development of reverse genetic tools to dissect host–parasite interactions as well as genome-guided interpretation of *Cuscuta* peptidome, secretome, and other sub-proteome data.

## Methods

### Plant material and DNA extraction

*C. campestris* was grown in a glasshouse at the phytotron of the University of Tromsø, Norway, in 24 h of light at 21 °C on *Pelargonium zonale* L. as host. DNA was extracted from stems and flowers using a modified protocol of Peterson et al.^40^. In brief, fresh plant material was incubated in the dark at 4 °C for 48 h to remove starch. Afterwards it was homogenized in extraction buffer (2-methyl-2,4-pentanediol (1 M), PIPES (10 mM), MgCl_2_ (10 mM), PVP-10 (4% w/v), sodium metabisulfite (10 mM), 2-mercaptoethanol (25 mM), sodium diethyldithiocarbamate (0.5% w/v), l-lysine (200 mM), and ethylene glycol tetraacetic acid (EGTA (6 mM), pH 6.0). The homogenized material was filtered twice through Miracloth, Triton X-100 was added to a total concentration of 0.5% (v/v) on ice and the sample was incubated for 30 min. Remaining starch was pelleted at 200 × *g* for 2 min. The supernatant was centrifuged at 800 × *g* for 20 min at 4 °C and the pellet was resuspended in 50 ml of MPDB (2-methyl-2,4-pentanediol (0.5 M), PIPES-KOH (10 mM), MgCl_2_ (10 mM), Triton X-100 (0.5% v/v), sodium metabisulfite (10 mM), 2-mercaptoethanol (5 mM), l-lysine (200 mM), and EGTA (6 mM, pH 7.0). Nuclei were pelleted at 650 × *g* for 20 min at 4 °C and then resuspended in MPDB buffer. Sodium dodecyl sulfate (SDS) was added to a final concentration of 2% (w/v). The sample was gently mixed and incubated at 60 °C for 10 min. Sodium perchlorate was added to final concentration of 1 M. After centrifugation at 400 × *g* for 20 min at room temperature, the supernatant was extracted twice with an equal volume of phenol/chloroform/isoamyl alcohol (25:24:1). DNA was precipitated by adding sodium acetate (0.3 M, final concentration) and two volumes of absolute ethanol at −20 °C, pelleted and washed with 70% ethanol (v/v).

### Genome size estimate

Genome size was measured by flow cytometry. Fresh *C. campestris* plant material was co-chopped together with the internal standard *Pisum sativum* (1C = 4.42 pg) in isolation buffer (citric acid (0.1 M), Triton X-100 (0.5% w/w), pH 1.5), filtered through a 30 µm nylon mesh, and treated with RNase A (0.15 mg ml^−1^). After staining with propidium iodide (50 mg l^−1^ in staining buffer), the suspension was measured with a CyFlow Space flow cytometer (Partec) up to a total of 10,000 particles. The results were calculated according to the formula 1C = (mean object fluorescence intensity/mean standard fluorescence intensity) * 1C of the standard. The results of three preparations were averaged.

### Cytology

Flower buds and open flowers from one individual plant were fixed in Carnoy’s fixative (ethanol: acetic acid, 3:1) for 24 h at room temperature and stored until use at −20 °C. Material was stained using the standard Feulgen staining technique. Briefly, material was rinsed with tap water, incubated for 20 min in HCl (5N) at room temperature, rinsed and stained with Schiff’s reagent (VWR) for 1 h. Preparations were analyzed with a Zeiss Axioplan microscope, and images were acquired with a digital CCD camera and Axiovision 4.8 software (Carl Zeiss). Chromosome number was established based on analyses of several preparations and at least five intact chromosome spreads.

### Genome sequencing

Three Illumina TruSeq PCR-free paired-end libraries with insert sizes of 550, 785, and 800 bp were prepared according to the manufacturer’s manual (Part# 15075699 Rev.A). Bead based size selection was applied to the first library, while BluePippin size selection with an internal R2 marker selecting from 700 to 930 bp was used for the two other libraries. DNA input was 4 µg, 8 µg, and 12 µg for libraries 01, 02, and 03, respectively. The remaining steps were conducted according to the Illumina protocol.

For the 4, 8, and 12 kb insert size Mate-Pair libraries the Nextera Mate Pair Kit (Illumina) was used with modifications. For the 4 kb insert size library two separate reactions with 4 µg of high molecular weight DNA were tagmented according to manufacturer’s instructions. The reactions were cleaned up separately using the Zymo Genomic DNA Clean & Concentrator Kit according to the Illumina Nextera Mate Pair manual and eluted to 30 µl each. The cleaned up tagmented DNA samples were then mixed with 10 µl loading solution (Sage Science), loaded on one lane of a Blue Pippin device (Sage Science) each and size selected from 2 to 6 kb each using the S1 marker (Sage Science). The samples were pooled after Pippin size selection and cleaned up using Ampure XP beads (Beckman Coulter) using an equal ratio of beads and sample. Afterwards strand displacement was carried out using the phi29 DNA polymerase (NEB) according to manufacturer’s instructions followed by another Ampure XP bead clean up with an equal ratio of beads and sample. The rest of the library preparation was carried out according to the Illumina Nextera Mate Pair protocol except using a BioRuptor Pico (Diagenode) for fragmentation with six cycles (5 s on, 60 s off) at 4 °C and a sample volume of 100 µl. The workflow was the same for the 8 kb insert size library except selection from 6 to 10 kb with the BluePippin device (Sage Science).

For the 12 kb insert size libraries size selection was carried out from 12 to 20 kb. In addition to the changes mentioned above, the tagmentation reaction was also modified to enrich for larger fragments. Instead of 12 µl, 8 µl of the Mate Pair Tagment Enzyme were used with a tagmentation buffer prepared as described earlier^41^. Three separate reactions were used with these volumes and pooled after size selection. Circularization time was extended to 70 h to allow self-ligation of larger fragments.

For 40 kb mate pair libraries, 15 µg of high molecular weight DNA was sheared using a Covaris g-Tube (cat no. 520079; Covaris) at 3000 rpm for 30 s in an Eppendorf 5415R centrifuge. The End-Repair was scaled up according to the NxSeq® 40 kb Mate Pair Cloning Kit protocol (Lucigen). Afterwards a 1× AMPure XP bead cleanup was performed. The end-repaired DNA was size selected using a 0.75% gel cartridge on high-pass protocol for selection of 40,000–80,000 bp on a BluePippin device. The sample was cleaned with 1 volume AMPure XP beads. All subsequent steps were performed according to the manufacturer’s protocol. All above described DNA libraries were sequenced using an Illumina MiSeq sequencer with either 600 cycle V3 (cat. no. MS-102-3003; Illumina) or 150 cycle V3 (cat. no. MS-102-3001; Illumina) chemistry. Illumina RTA version 1.18.54 was used for basecalling and the generation of fastq files with deactivated adapter trimming.

### PacBio sequencing

Long read sequencing using the PacBio RS system was performed by the commercial sequencing provider GATC Biotech. Three SMRT cells yielded a total of 493,246 subreads representing 4.16 Gbp of sequence data (Supplementary Fig. 11). The data with a maximal read length of 48.8 kb and a read length N50 of 12 kb equates to >8-fold genomic coverage. PacBio subreads were deduplicated, by only keeping the longest subread in each case. The resulting, deduplicated set was then used for scaffolding only.

### Genome assembly

All Illumina data was trimmed for low-quality bases and adapter sequences using Trimmomatic v0.35^42^. Illumina paired-end TruSeq PCR-free libraries were filtered using standard settings, while Mate-pair libraries were further filtered for the transposase sequence. For all libraries quality filtering used a sliding window of 4:15 and a minimal read length of 36 bp.

Insert sizes were estimated by aligning the filtered reads to the contig assembly as unpaired using bowtie2 (v2.2.6)^43^. The mapping position of reads aligned to the same contig in the expected orientation was used to calculate the insert size.

Two fosmid libraries were prepared according to the Lucigen 40 kb Mate Pair cloning kit and sequenced on an Illumina MiSeq sequencer. For the deduplication of fosmid data, the reads were processed with Trimmomatic using standard settings^42^. Unpaired reads were discarded and paired reads were subsequently filtered for presence of the “CAC” sequence at the start of both forward and reverse read as well as the “GTAC” restriction enzyme motif. If the motif was found, the read was trimmed accordingly. Only reads with 20 bp or longer were kept. These were aligned to the assembly as unpaired using bowtie2 (v2.2.6) with standard settings. The alignment was used to identify duplicates based on identical mapping positions and discarding ambiguous alignments based on a mapping quality greater than 3. Valid intralinks were checked for correct orientation and used for the insert size estimation.

For the deduplication of Illumina mate-pair data, all mate-pair libraries were de-duplicated based on the mapping position analogous to the fosmid libraries without initial filtering of the Lucigen fosmid specific “CAC”- and restriction motif.

Genome characteristics were estimated using a k-mer approach as described^44^. A total of 31 billion 19-mers were created from the 149.11 million filtered paired-end reads using Jellyfish^45^. The respective 19-mer depth was plotted to determine the peak k-mer depth of 54. Given the local minimum of the 19-mer depth distribution at a 19-mer depth of 17, a total of 34 million 19-mers were considered to be the result of sequencing errors. Dividing the remaining 30 billion 19-mers by the determined peak 19-mer depth of 54 resulted in a genome size estimate of 557 Mb.

The trimmed Illumina DNA paired-end data was assembled into contigs using DISCOVAR de novo r52488^46^. Resulting contigs were used for assembling scaffolds with SSPACE-Standard v3.0^47^ using the de-duplicated mate-pair and fosmid libraries. Scaffolds greater than 1000 bp were kept and scaffolded further with PacBio data using SSPACE-Longread v1.1^47^. At least five links were required in both scaffolding steps for contig pairing. Gaps in the assembly were filled with GapFiller v1.10^48^ with all Illumina paired-end and mate-pair data but omitting the PacBio data. Subsequently, lowly supported regions on scaffolds were identified and scaffolds split.

### Chloroplast genome assembly

The chloroplast genome (Supplementary Fig. 10) was assembled using a high-coverage subset of the 7.59 M filtered paired-­end reads of the TruSeq PCR­free 800 bp insert library^45^. This high coverage subset should be enriched in chloroplast reads, since the chloroplast genome has many copies per cell. To determine which reads came from high coverage regions, the 19-mer coverage of the library was determined and the average 19-­mer coverage was estimated for each read based on the library 19­-mer distribution. Read pairs indicating a 19­-mer coverage greater than 250× were kept and assembled using SPAdes v.3.6.1^49^ without further error correction. The assembly yielded a total of 29,261 contigs ranging from 128 to 51,323 base pairs. Within the 50 longest contigs, three contigs showed a higher coverage of 266, 382, and 431, respectively, as estimated by SPAdes. Orientation and order of the contigs were built manually by aligning the reads back to the assembly using bwa v0.7.5a­r418^50^. For confirmation and to identify the overlapping sequences, all three contigs were searched against each other using BLASTn v2.2.27+ with default settings. Contigs associated to the assembled chloroplast genome were removed from the whole-genome assembly based on alignments using BLAST.

### Contaminant removal

For the removal of contaminants, the assembly was aligned using a BLAST search against the non-redundant nucleotide NCBI BLAST database. Additionally, all scaffolds were aligned using BLAST against the Enterobacter phage phiX174 reference (NC_001422.1), and the *Pelargonium* x *hortorum* chloroplast reference (DQ897681.1). Only the BLAST against *Pelargonium* indicated full-length hits against 25 small scaffolds that were subsequently excluded from the assembly.

### Genome completeness assessment and gene annotation

Assessment of genome and gene-set completeness was quantified using BUSCO v3.0.1^11^ using the embryophyta_odb9 single-copy orthologs and default settings. Gene annotation was performed in a multiple-step process. RNAseq data was first used to create draft gene models, which were manually checked, and used to train Augustus. Then RNAseq data and homology data were used as extrinsic evidence to guide de novo gene prediction with Augustus. Finally, predicted genes were post filtered as described below to create a high confidence gene set.

### RNAseq and gene model creation

RNAseq was performed on three replicates each of non-infectious feeding stems, detached starving stems and different infection stages to cover all important situations in the parasites life. Samples were sent to GATC (Germany) for sequencing using Illumina MiSeq. Read quality was checked with FastQC v0.11.2 (www.bioinformatics.babraham.ac.uk/projects/fastqc) and trimmed using Trimmomatic, version 0.32^42^. TruSeq3PE-2 adapters and the overrepresented k-mer TATATACTAG, were trimmed allowing three seed mismatches, and a palindrome and simple clip threshold of 30 and 10, respectively. Very low quality (below phred 3) bases were trimmed from both ends of read, and quality trimming was completed with Trimmomatic’s MAXINFO tool with target length of 50 and strictness of 0.8.

### Repeat masking

Repetitive genomic regions were determined with RepeatMasker using the *Arabidopsis* repeat element database. Hard masking was used for mapping of RNAseq data and cross-species protein mapping, while soft masking was used during final gene calling and quality control.

### Mapping

Trimmed RNASeq reads were mapped with TopHat v2.0.14^51^ using the very sensitive bowtie2 setting.

### Training augustus

Cufflinks v2.2.1^52^ was used to create draft transcript models (settings: min-frags-per-transfrag 100, max-multi-read-fraction 0.5), and Cuffmerge was used to combine the models from each library. Proteins were predicted from these using TRAPID^53^ with orthoCML 5 database as reference, and filtered to those at least 30 amino acids long. From these gene models, a training set was defined of 679 genes (529 to train, 150 to test) that (i) had three-way best BLAST hit between *C. campestris* draft, *A. thaliana*^54^, and *S. lycopersicum*^12^; (ii) these three orthologs shared a start codon, and were all within 20 bp in aligned length; (iii) the *C. campestris* draft genes shared no more than 60% protein sequence homology with another gene in the set; and (iv) start codon of protein was successfully mapped back to genome with Exonerate version 2.2.0^55^. The intergenic region half-way to the next cufflinks gene model was included as genomic background for training Augustus, and training was performed with UTR on.

### Providing extrinsic data as hints to augustus

Coverage hints for Augustus were generated with rsem-bam2wig (settings: no-fractional-weight^56^) and the Augustus wig2hints script (settings: prune = 0.1, type = exonpart, width = 10, margin = 10, minthresh = 2, minscore = 240, src = W, radius = 4.5). Intron hints were generated with the Augustus bam2hints script (settings: intronsonly).

Homology hints were obtained by mapping *A. thaliana*, *S. lycopersicum*, and all entries matching “Cuscuta” from NCBI’s protein database to the genome with Exonerate (settings: model = protein2genome, maxintron = 30000, showtargetgff = T, and percent = 40/50/60 for respective species). Mappings were converted to hints with the Augustus script exonerate2hints.pl (settings: CDSpart_cutoff = 0, and priority *Cuscuta* > *S. lycopersicum*, *A. thaliana*).

### Gene calling

Augustus was configured to heavily weight extrinsic evidence (Supplementary Data 3) and run with the following settings (alternatives-from-evidence = true, alternatives-from-sampling = true, UTR = on, strand = both, minexonintronprob = 0.3, minmeanexonintronprob = 0.6, maxtracks = 2, softmasking = 1). Finally, high confidence gene models were defined as those fulfilling having 10 or more hits to the nr database at *e*-value < 10^−8^ and/or expressed with at least 100 unique counts (~0.18 reads per million mapped reads) between all RNAseq samples (calculated with HTSeq-0.6.1^57^). This dual filter excludes only genes that are neither clearly expressed nor homologous to known genes in other species.

### Gene expression analysis

Trimmed RNASeq reads were mapped as for gene calling above, except that the new gene models were provided to TopHat to facilitate mapping across identified splice junctions (-G gene_models.gff). Subsequently, unique counts were quantified using HTSeq-0.6.1 using default parameters expect for --stranded = no. Paired and single reads were counted separately and summed as fragments using cufflinks v2.2.1 using the -G setting. Differential gene expression analysis was performed with edgeR.

### Transposon composition

Transposons were detected and classified by a homology search against the REdat_9.8_Eudicot section of the PGSB transposon library^58^ which had been complemented beforehand by 974 non-redundant *C. campestris* specific full length LTR retrotransposons as described below to identify fragmented and partial copies. The program vmatch (http://www.vmatch.de), a fast and efficient matching tool, was used with the following parameters: identity≥70%, minimal hit length 75 bp, seedlength 12 bp (exact commandline: ­d ­p -l 75 ­identity 70 ­seedlength 12 ­exdrop 5). The vmatch output was filtered for redundant hits via a priority based approach, which assigns higher scoring matches first and either shortens (<90% coverage and ≥50 bp rest length) or removes lower scoring overlaps to obtain an overlap free annotation. Full-length LTR retrotransposons elements were identified with LTRharvest^59^, which reported 7012 non-overlapping candidate sequences under the following parameter settings: “overlaps best -seed 30 -minlenltr 100 -maxlenltr 2000 -mindistltr 3000 -maxdistltr 25000 -similar 85 -mintsd 4 -maxtsd 20 -motif tgca -motifmis 1 -vic 60 -xdrop 5 -mat 2 -mis −2 -ins −3 -del −3”. All candidates were annotated for PfamA domains with hmmer3^60^ (http://hmmer.org/) and stringently filtered for false positives by the following criteria: (1) presence of at least one typical retrotransposon domain (RT, RH, INT, GAG), (2) removal of hybrids with inconsistent domain mixes e.g. RT, RH, INT, RT, RH, (3) absence of gene Pfam domains, (4) strand consistency between domains and primer binding site, (5) tandem repeat content below 25%, (6) long terminal repeat≤25 of the element length and (7) an *N* content < 5%. The inner domain order was used for the subfamily classification into Ty3/*gypsy* (RT-RH-INT), Ty1/*copia* (INT-RT-RH) or unknown if either INT or RT were missing. The filtering steps led to a final set of 974 high confidence full-length LTR retrotransposons, which were clustered with a >95% identity over >95% mutual coverage to a non-redundant set of 969 elements.

LTR retrotransposons (8–20 kb) have the special structural property of LTRs (~1–2 kb) at both ends, which are identical upon insertion because only the 5′ end serves as a template. This feature enables a dating of the insertion event by comparing the divergence between the 5′ and 3′ LTRs^61^. A random mutation rate of 7*10^−9^ was used to convert the sequence divergence into age^62^.

### Orthogroup and functional enrichment analysis

Orthogroups were created with OrthoFinder^63^ from proteins of primary transcripts of the *C*. *campestris* high-confidence set, *Ipomoea* nil, *Solanum* *lycopersicum*, *Amaranthus hypochondriacus*, *Arabidopsis thaliana*, *Daucus carota*, *Mimulus guttatus*, *Oryza sativa*, *Sorghum bicolor*, *Vitis vinifera*, *Populus trichocarpa*, and *Dioscorea rotundata*. Sequences for additional species were obtained from Phytozome v12.0 (https://phytozome.jgi.doe.gov), except I. nil from ftp://ftp.ncbi.nlm.nih.gov/genomes/Ipomoea_nil/, and *D. rotundata* from http://genome-e.ibrc.or.jp/home/bioinformatics-team/yam. Functional categories were assigned to each *A. thaliana* gene using Mercator. Fisher’s exact test was used to test for enrichment of functional categories (based on the function of all the *A. thaliana* genes in each orthogroup) in orthogroups without a *C. campestris* gene. Specifically, to characterize gene loss a comparison was performed between 0:1 (or more) and remaining orthogroups (*C. campestris*: each other dicot species; Fig. 3c). Complementarily, to characterize gene duplication, for orthogroups with at least one gene in every dicot species, a comparison was performed between orthogroups with a ratio of at least 2*n*:*n* (*C. campestris*: each other dicot species; Fig. 3c) and the rest. The false discovery rate was calculated according to the Benjamini and Yekutieli procedure^64^.

### Synteny analysis

Syntenic regions and tandem duplicates were identified with MCScanX^65^ using stricter than default settings (*e*-value 10^−20^, maximum gaps 15, minimum size of collinear block 8). The protein BLAST results from Orthofinder were used as input. MCScanX was ran once on the individual species *C. campestris* and *A. thaliana* to classify paralogs, and once with *C. campestris* and the high quality reference assemblies of *S. lycopersicum* and *A. thaliana* together to determine cross-species ratios.

### Substitution rate analysis

The synonymous substitution rate (d*S*) was calculated for 1:1 orthologs (other species: *C. campestris*) and paralogous pairs (exactly two genes in orthogroup for *C. campestris*). Protein alignment was performed with PRANK^66^, converted to nucleotide alignment with pal2nal^67^, and dS was calculated with codeml from PAML^68^. All of the above was orchestrated via the wrapper program phasePAML (https://github.com/janinamass/phasePAML).

### Comparative analysis of gene losses across the plant kingdom

A comprehensive search for gene losses in *C. campestris* was performed using *I. nil*, a non-parasitic plant within the same taxonomic family of Convolvulaceae, as reference. Protein sequences from *I. nil*^14^ were tested against a comprehensive set of protein sequences from more than hundred Angiosperm species (Supplementary Data 4) to select all proteins that were conserved within or beyond the order Solanales using a BLASTp search with score values > 50. The 23,700 conserved candidate protein sets were analyzed for the presence of homologs in *Cuscuta* using our *C. campestris* protein models as well as the predicted proteins from the transcriptomes of *C. pentagona* and *C. suaveolens*^8, 69^. The protein sets without significant *Cuscuta* sp. homologs were used to generate multiple sequence alignments. Hidden Markov Models (HMM) constructed from these multiple alignments were used to verify the absence of homologous *Cuscuta* sp. proteins. Finally, 1736 proteins from *I. nil* without significant *Cuscuta* sp. homologs were confirmed.

Gene presence or absence in 14 dicotyledonous angiosperms, two monocotyledonous angiosperms, one moss and two Chlorophyte species was also inferred for a selected set of proteins (Fig. 4a) using a multistep BLASTp analysis. All amino acid sequences were derived from available whole-genome data sets, published without restrictions on Phytozome v12.0.

### Identification and verification of HGT candidates

HGT candidates were identified using an “extended best hit” approach that allows eliminating false or dubious HGT results. First, BLASTp-analysis of all translated primary transcripts of the *C. campestris* high-confidence set was performed against the nr database with hit parameters limited to 25 hits per protein and an *e* value of 1e^−5^. The BLAST results for each protein were assigned to taxonomic bins based on the hit scores using the MEGAN v.6.0.0 software^70^ using the following parameters: minSupport = 1, minScore = 50, maxExpected = 0.01, minPercentIdentity = 0, topPercent = 40, lcaPercent = 100, minComplexity = 0, paired reads = false, useIdentityFilter = false. The topPercent value is a relative cutoff value that determines which bitscore, relative to the best-hit bitscore, any hit must have in order to be counted as part of the taxonomic bin containing the entire set (Supplementary Fig. 4). *C. campestris* proteins that were assigned to orders other than Solanales, were considered potential HGT candidates and were further subjected to filtering to obtain a high confidence set. The filtering steps included the removal of all proteins with a best hit bitscore of 80 or lower, as lower values compromised the topPercent = 40% rule, and a removal of sequences identified as transposable or repetitive elements and retrovirus sequences. Also, all sequence alignments were inspected manually and cases, where a drop in bitscores was due to a short inhomologous insertion that led to two instead of one alignments with lower bitscores each, were discarded. All HGT candidates were analyzed using the gene tree—species tree reconciliation approach of Notung^24^ to get further proof for transfer events. This approach relies on a comparison of gene trees with the corresponding species trees and the identification of deviating placements of the gene of interest in both trees. For the species trees, orthologous sequences for 350 randomly selected amino acid sequences from *C. campestris* were identified by BLASTp (*e*-value 10^−5^, max_target_seqs 1) in 41 plant species representing different branches of the phylogenetic tree. The minimum length of the random sequences was 200 amino acids. For each of these orthologous sequence sets a sequence alignment and a phylogenetic tree was generated using Clustal omega (auto) and FastTree 2^71^. The resulting trees were then analyzed by ASTRAL-II^72^ to generate a highly supported species tree (Supplementary Fig. 5). Some of the randomly picked polypeptides were not found in all taxa, leading to trees with “missing” branches in the trees. Only trees with at least 39 of the expected 42 branches were used. The analysis was repeated four times with similar results.

For the computation of HGT candidate gene trees, orthologous sequences of the 73 translated HGT candidates of *C. campestris* (7 albumin genes, 2 strictosidine synthase-like genes, and 64 novel candidates, see Supplementary Table 4) were identified by BLASTp (*e*-value 10^−5^). For each candidate, potential donor scenarios were reconstructed through reconciliation of the respective gene tree with the species tree using Notung 2.9^24^.

### Data availability

Raw reads, the assembled genome sequence and annotations are available from the European Nucleotide Archive (ENA) under accession number PRJEB19879 and from the plaBi database (http://plabipd.de/portal/cuscuta-campestris). Sequence alignments are deposited in the Open Science Framework (https://osf.io/KQD9V/) (10.17605/OSF.IO/KQD9V).

## Electronic supplementary material

Supplementary Information

Description of Additional Supplementary Files

Supplementary Data 1

Supplementary Data 2

Supplementary Data 3

Supplementary Data 4

Supplementary Data 5
