# Supplementary material for: Footprints of parasitism in the genome of the parasitic flowering plant Cuscuta campestris
Source: Nat Commun. 2018 Jun 28;9:2515. doi: 10.1038/s41467-018-04344-z (PMC6023873; doi:10.1038/s41467-018-04344-z)
Supplement: Supplementary file 1 — Supplementary Information [file 41467_2018_4344_MOESM1_ESM.pdf]

SUPPLEMENTARY INFORMATION

Footprints of parasitism in the genome of the parasitic flowering plant *Cuscuta campestris*

Vogel et al.

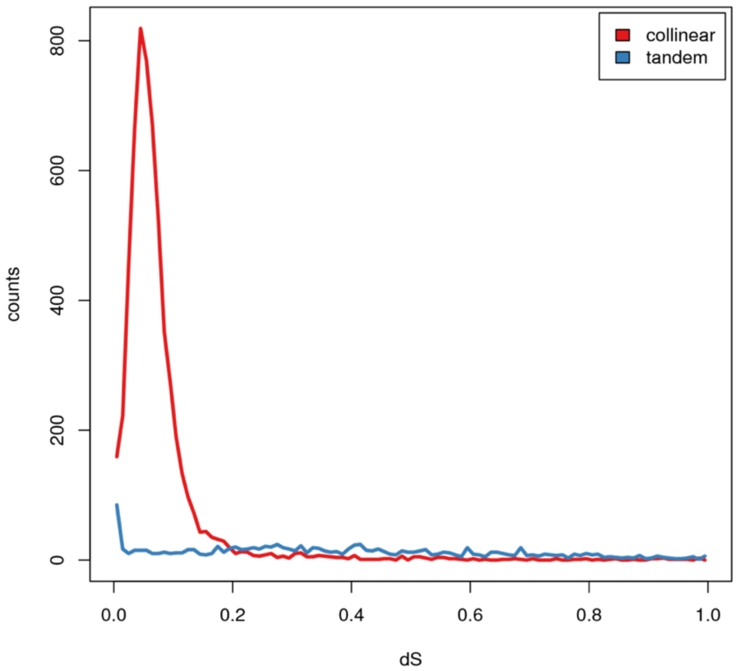

Supplementary Fig. 1 Paralog ds rate analysis. Synonymous substitution rate (dS) analysis; density of dS rates of *C. campestris* paralogs classified as collinear (syntenic) or tandem by MCScanX. Only simple pairs (exactly two genes) were counted for simplicity.

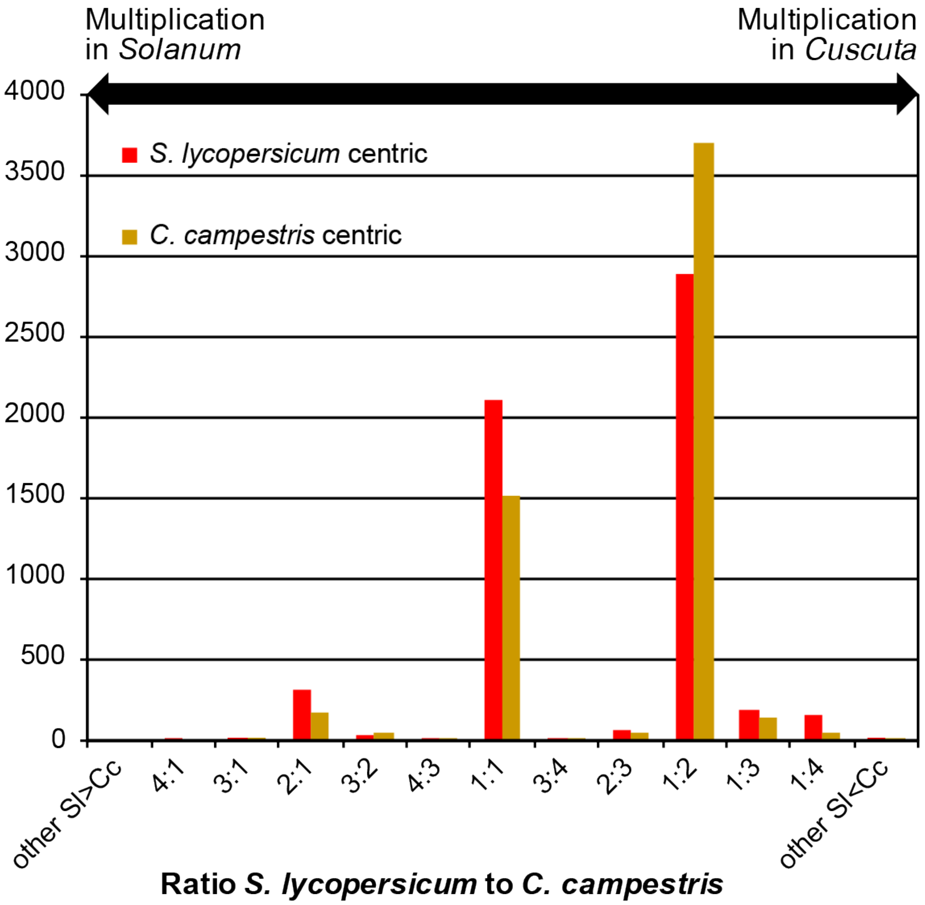

Supplementary Fig. 2 Homolog analysis in syntenic blocks between *S. lycopersicum* and *C. campestris.* Ratio of homologs occurring in syntenic blocks between *S. lycopersicum* and *C. campestris*. Number of syntenic homologous gene groups when quantified from MCScanX output alignment that is *C. campestris* centric (gold) or *S. lycopersicum* centric (red).

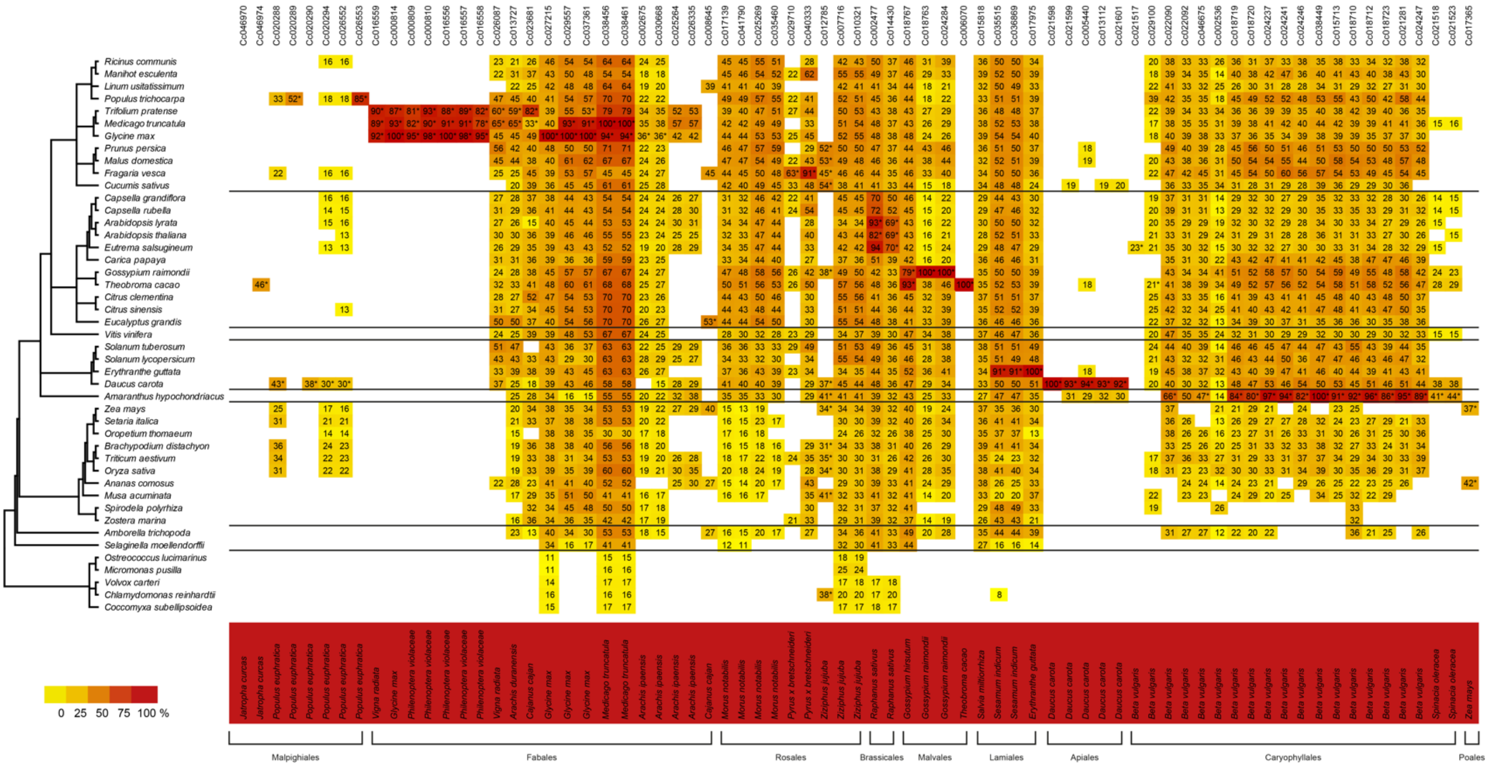

Supplementary Fig. 3 Sequence similarity of HGT candidates in *C. campestris* with orthologs from species with sequenced genomes. Amino acid sequences of HGT candidates (shown on top) compared against the genomes of 45 species from the plant kingdom (left) using BLASTp. The heat map shows the bit scores in each species as a percentage of the best-hit bit score from a BLASTp-search using the NCBI nr database (shown in Extended Data Table 3). The species with the best hit that represents the 100% reference value is shown at the bottom in red. A linear colour scale from yellow (0%) to red (100%) was used as visual aid in the table. Asterisks in the columns highlight potential HGT donors or their available ancestors as suggested by a Notung 2.9 based analysis.

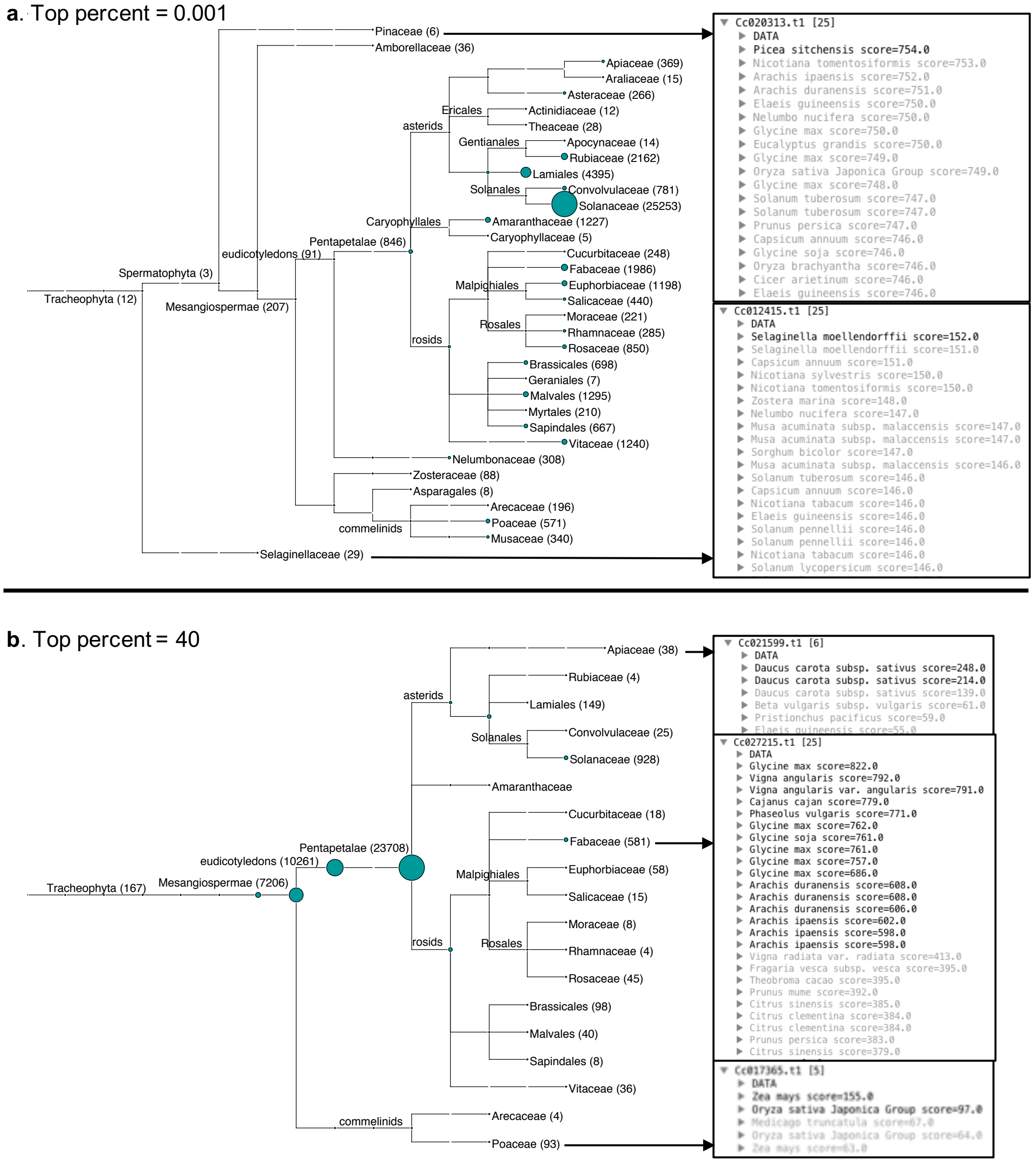

Supplementary Fig. 4 Taxonomic profiling of (**a**) best hits and (**b**) extended best hit lists obtained by BLASTP-comparison of *C. campestris* amino acid sequences to the non-redundant protein database (nr) from NCBI using MEGAN6[1]**.** Taxon resolution was set to families. Circles at the nodes correspond in size to the number of hits obtained for the respective taxon (shown in brackets). The absolute best hit bitscore cutoff was 50. **a** A relative cutoff of 0.001% of best hit bitscore allows for a best-hit-only evaluation independent of how conserved a sequence is across different taxa. **b** A relative cutoff of 40% of best hit bitscore places conserved sequences into higher phylogenetic ranks (phylum or class), while faster-evolving or taxon-specific sequences are assigned to lower phylogenetic ranks (e.g. family or genus). Framed boxes on the right show screen shots of the hit inspection window, where hits that count towards the taxon rank assignment are shown in black and hits that were not considered according to the cutoff settings are shown in grey (only best hit for (**a**) and all hits within 40% of top hit for (**b**)).

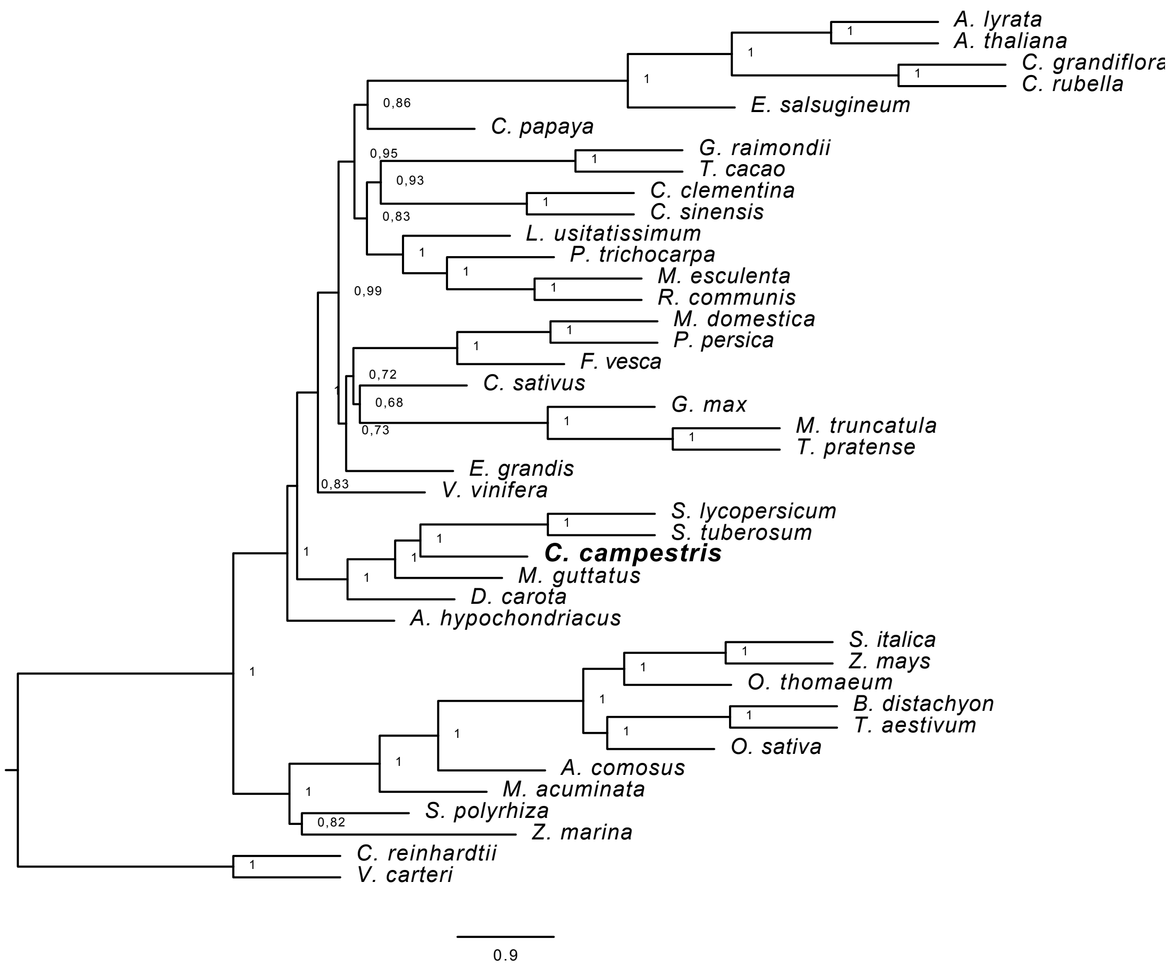

Supplementary Fig. 5 Phylogenetic placement of *C. campestris* as calculated with Astral-II[2]. The tree is based on 350 random amino acid sequences (min length 200) of *C. campestris* (in bold font) and their ortholog sequences of 41 plant species. Numbers indicate the branch support values, where one is the highest support. The scale bar indicates the coalescent units of the branches, which is a direct measure of the discordance in the gene trees.

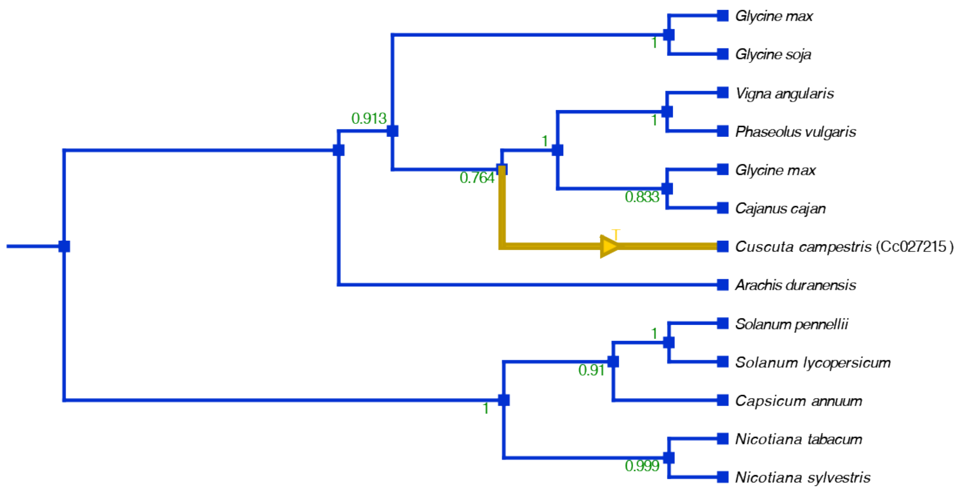

Supplementary Fig. 6 Prediction of gene donors for the HGT candidate gene shown in Fig. 5b. The yellow arrow indicates the predicted direction of the transfer. Numbers at the nodes are edge weight values determined by Notung[3] based on local support values estimated by Fasttree[4].

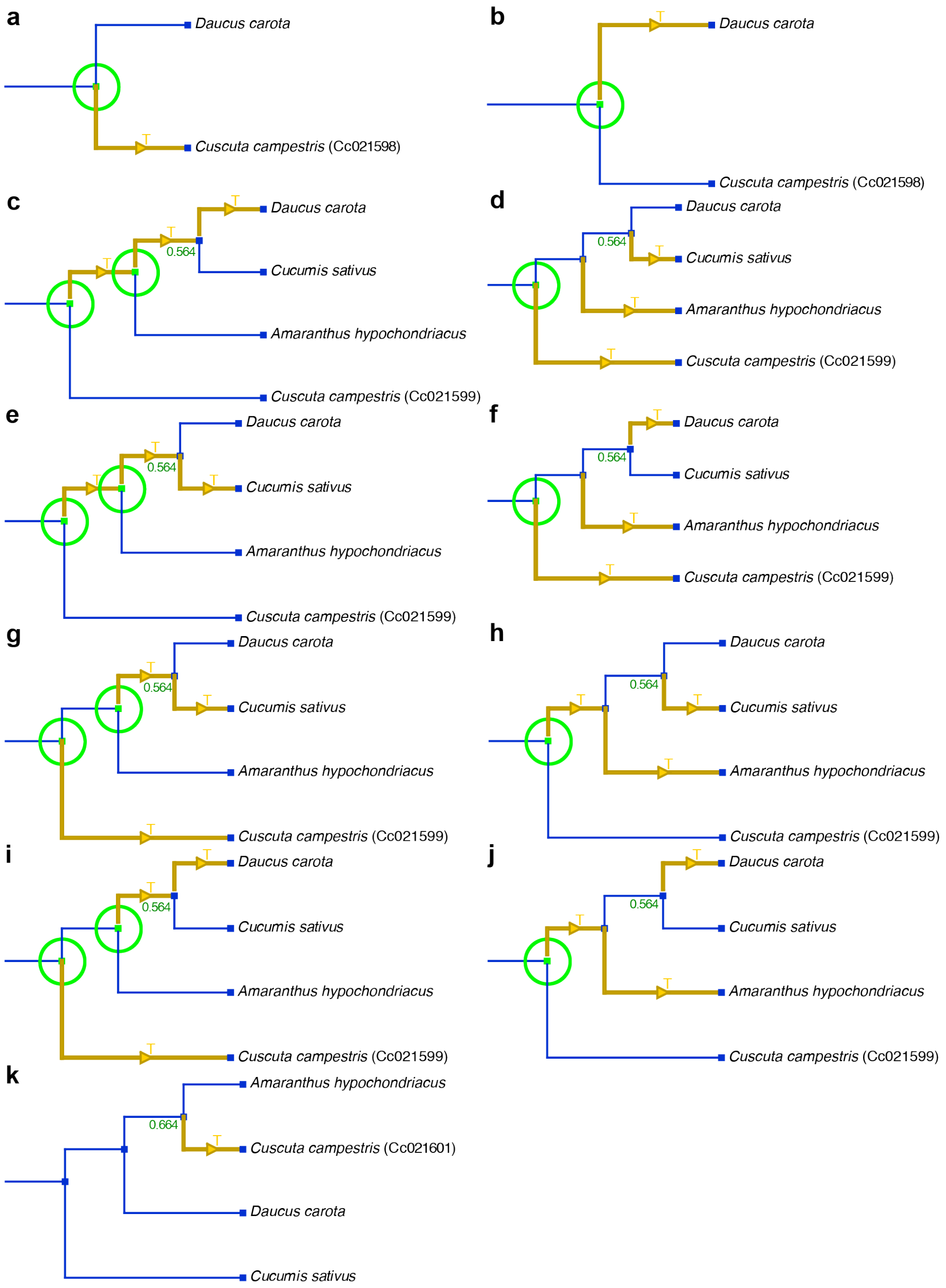

Supplementary Fig. 7 Alternative HGT event solutions predicted by Notung 2.9 for three genes located on the putatively transferred DNA fragment shown in Fig. 5c. **a, b** Two solutions offered for *Cc021598*. **d - j** Eight solutions offered for *Cc021599*. **k** One solution offered for *Cc021601*. Yellow arrows indicate predicted directions of the transfer. Green circles indicate different solutions of the reconciliation of the respective gene tree with the species tree (Supplementary Fig. 5). Numbers at the nodes are edge weight values as determined by Notung[3] based on local support values estimated by Fasttree[4].

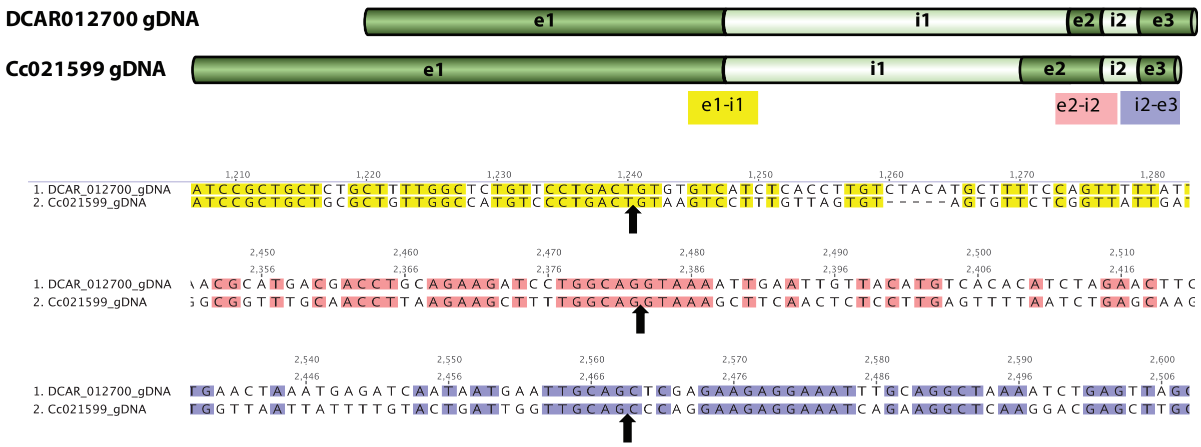

Supplementary Fig. 8 Conservation of intron positions in HGT candidate *Cc021599* and its closest ortholog from *D. carota*. On top, a schematic overview of the three exons (e1-3, in dark green) and two introns (i1-2, in light green) of both genes are shown. Below, sequences surrounding the conserved borders between exon 1/intron 1 (in yellow), exon 2/intron 2 (in red) and intron 2/exon 3 (in blue) are depicted. Sequence identities between both genes are highlighted in with the respective colors. The exact border between exon and intron (or vice versa) are indicated with arrows.

Cuscuta campestris	HR-1dpi vs. FS	HR-3dpi vs. FS	Predicted function or domain
Cc038461	-3.190	-5.978	Clan71/CYP76-type cytochrome P450 monooxygenase
Cc037361		-5.749	Anthocyanin malonyl-transferase, BAHD-type
Cc038456	-2.315	-4.034	Clan71/CYP76-type cytochrome P450 monooxygenase
Cc000810	-1.607	-1.978	Albumin
Cc013112	-0.597	-1.157	Uncharacterized protein, Kelch motif
Cc021599	-0.758	-1.139	Uncharacterized protein, Kelch motif
Cc016557		-1.091	Albumin
Cc016558		-0.935	Albumin
Cc000809		-0.905	Albumin
Cc030668		-0.687	Ankyrin repeat
Cc006070		-0.599	Uncharacterized protein
Cc027215	0.949		CES101 G-type lectin receptor-like kinase
Cc022090	0.928	0.481	hypothetical or uncharacterized protein
Cc020289	0.499	0.623	hypothetical or uncharacterized protein
Cc017139		0.655	F-box protein
Cc035460		0.687	F-box protein
Cc024237		0.696	F-box protein CPR30-like
Cc021517	0.526	0.716	Uncharacterized protein
Cc046675	0.530	0.819	F-box/Kelch repeat
Cc025264	0.854	0.923	Replication factor-A CTD-like
Cc015713	0.728	0.991	F-box protein CPR30-like
Cc020290	0.714	1.122	hypothetical or uncharacterized protein
Cc007716	3.710	1.156	Receptor kinase
Cc026553	1.138	1.410	hypothetical or uncharacterized protein
Cc021601	0.875	1.438	Uncharacterized protein. Kelch motif
Cc029710	1.379	1.495	Zinc finger BED domain containing protein
Cc010321	3.879	1.616	Receptor kinase
Cc026552	1.337	1.683	hypothetical or uncharacterized protein
Cc017975	1.724	2.127	Late blight resistance protein homolog RIA-3
Cc023681	2.183	2.213	FAR1-related seq. 5
Cc020294	2.006	2.245	hypothetical or uncharacterized protein
Cc015818		5.200	lectin-like receptor kinase SMLII
Cc018720	4.446	6.827	F-box protein CPR30-like
Cc035515	7.523	7.133	Xyloglucan-specific endoglucanase inhibitor
Cc036869	5.832	7.369	Xyloglucan-specific endoglucanase inhibitor

-6 -1 1 7

Supplementary Fig. 9 HGT candidates showing differential expression during haustorial induction. Fold-changes ($\log_2$) in gene expression are shown for stems where haustoria were induced by far-red light (HR)[5], 1 or 3 days post induction (dpi) in comparison to non-infectious feeding stems (FS). The FDR cut-off was set to <0.05 (Supplementary Data File 2). A linear color scale from yellow (-6) to blue (7) was used as visual aid in the heatmap.

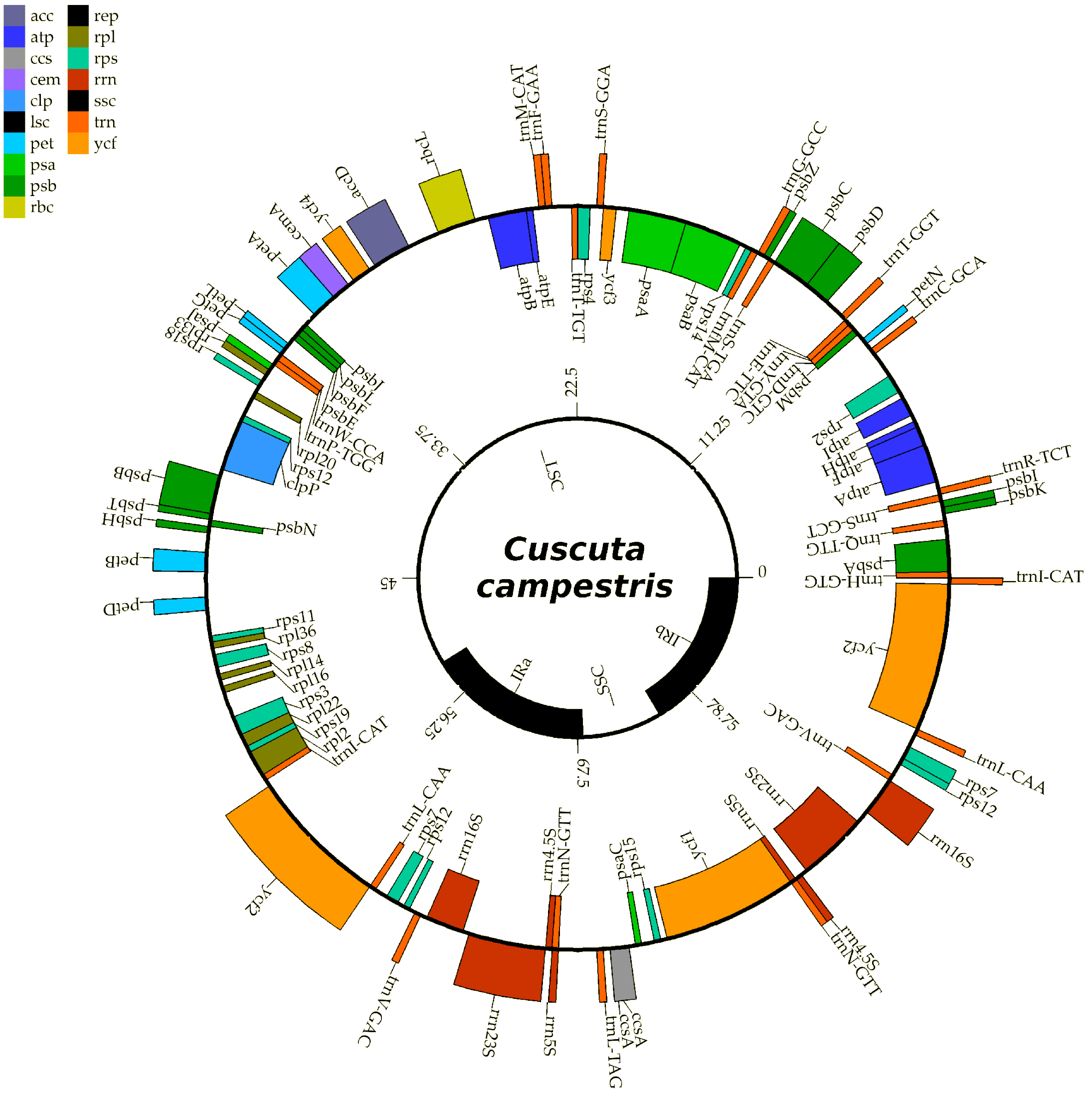

Supplementary Fig. 10 Circular map of the plastid genome. The map was drawn with CpGAVAS[6] using the plastid genome assembly described in Online Methods. Genes on the outside of the outer ring are transcribed counter-clockwise, genes on the inside clockwise. Positions of the inverted repeats and the large and small single copy regions are indicated on the inner ring. Colors decode different functional groups of genes.

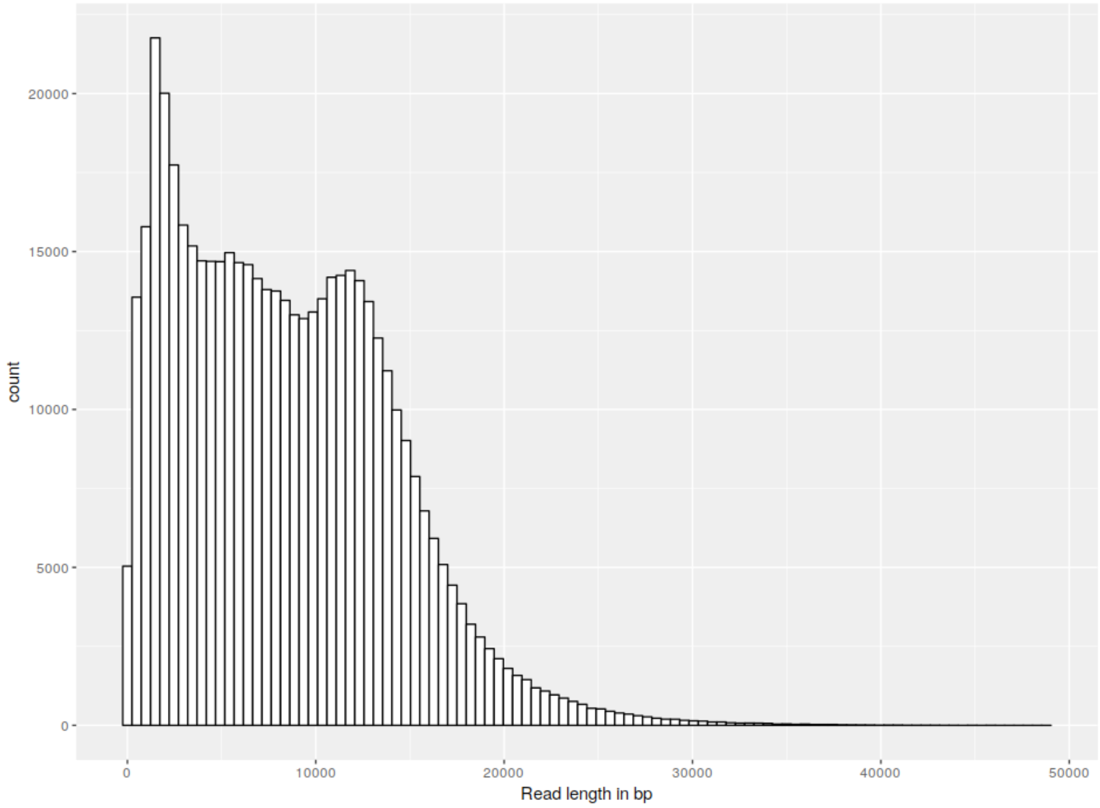

Supplementary Fig. 11 Sub-read length distribution for *C. campestris* PacBio data. Read length distribution for all 493,246 PacBio sub-reads comprising 4.16 Gbp of sequence data with a maximal observed read length of 48.8 kb. The data represents approximately 8.3-fold genomic coverage.

Supplementary Table 1 Genome statistics for *C. campestris*.

Stage	Contigs /Scaffolds	Total size (bp)	N's	Longest scaffold (bp)	Shortest scaffold (bp)	N75 (bp)	N50 (bp)	N25 (bp)
**Contigs**	591,173	1,064,390,962	0	173,761	200	5,001	16,161	31,215
**Paired-end**	254,705	524,962,404	5438	347,423	200	7,082	27,909	58,521
**4 kb Mate-Pair**	231,798	536,634,835	12,775,187	948,39	200	32,773	99,108	203,758
**8 kb Mate-Pair**	227,459	544,966,668	21,133,051	3,066,895	200	75,963	228,62	492,831
**>12 kb Mate-pair**	225,883	548,038,478	24,219,260	3,436,115	200	140,036	433,539	910,46
**40 kb Fosmids**	10,151	487,105,279	29,346,872	5,325,031	1,001	527,628	1,098,382	2,273,996
**PacBio Scaffolding**	6,768	476,654,732	33,100,169	5,338,161	1,001	638,838	1,461,197	2,877,936
**Gap Filling**	6,768	476,963,533	26,220,512	5,342,339	1,001	639,229	1,462,163	2,880,294
**Assembly validation**	6,907	476,794,675	26,064,366	5,342,339	1,001	577,005	1,384,808	2,799,340
**Final**	6,907	476,794,675	26,064,366	5,342,339	1,001	577,005	1,384,808	2,799,340

Statistics of the genome assembly at various stages are given, where the individual stages are given in the first column.

Supplementary Table 2 Gene content completeness assessment.

	S. lycopersicum	*S. tuberosum*	*I. nil*	*I. trifida*	*C. campestris*
**Complete**	1387 (96.3 %)	1395 (96.8 %)	1349 (93.7 %)	1325 (92.1 %)	1182 (82.1 %)
**Single-Copy**	1362 (94.6 %)	1357 (94.2 %)	1244 (86.4 %)	1242 (86.3 %)	333 (23.1 %)
**Duplicated**	25 (1.7 %)	38 (2.6 %)	105 (7.3 %)	83 (5.8 %)	849 (59.0 %)
**Fragmented**	12 (0.8 %)	11 (0.8 %)	27 (1.9 %)	43 (3.0 %)	25 (1.7 %)
**Missing**	41 (2.9 %)	34 (2.4 %)	64 (4.4 %)	82 (4.9 %)	233 (16.2%)
**Total**	1440	1440	1440	1440	1440

To assess the gene content the *C. campestris* genome and related genomes were analyzed using BUSCO[7].

Supplementary Table 3 Repetitive elements in *C. campestris.*

	% of genome	% of TE base pairs	number	MB
Mobile Element (TXX)	46.2	100	689,133	220.5
Class I: Retroelement (RXX)	44.9	97.1	673,098	214.1
LTR Retrotransposon (RLX)	44.5	96.4	669,593	212.5
Ty1/*copia* (RLC)	15.8	34.1	201,670	75.3
full-length RLC	0.4	0.9	242	1.9
Ty3/*gypsy* (RLG)	7.8	16.9	103,144	37.2
full-length RLG	0.4	0.8	204	1.8
unclassified LTR (RLX)	21.0	45.3	364,779	100.0
full-length RLX	0.6	1.2	248	2.7
non-LTR Retrotransposon (RXX)	0.3	0.8	3,505	1.7
Class II: DNA Transposon (DXX)	1.3	2.7	12,576	6.1
DNA Transposon Superfamily (DTX)	1.2	2.5	7,163	5.6
MITE (DXX)	0.1	0.2	5,413	0.5
Unclassified Element (TXX)	0.1	0.1	3,459	0.3
Retro-TE/DNA-TE ratio	*35.4*			
Copia/Gypsy ratio	*2.0*			

Detection method: vmatch versus REdat_9.8_Eudicot. Transposons were identified by homology to a customized Eudicot transposon library, which had been complemented with de novo detected *C. campestris* full length LTR-retrotransposons (details in the method section). Additional estimates for *C. campestris* specific non-LTR and DNA transposons missing in the transposon library are based on the occurrence of typical Pfam domains for these transposon types.

Supplementary Table 4: Horizontal gene transfer candidates.

Cuscuta protein		Best hit in NCBI nr database			Taxon containing all hits within relative cutoff (40% of best hit)		Predicted function or domain	MapMan category
#	amino acids	Bit-score	NCBI Acc. #	Species	Taxon	Order		
Cc046970	361	117	KDP34176.1	*Jatropha curcas*	Jatropha	Malpighiales	hypothetical or uncharacterized protein	unknown
Cc046974	421	103						
Cc020288	158	208	XP_011001738.1	*Populus euphratica*	Populus			
Cc020289	334	213						
Cc020290	182	187						
Cc020294	436	375						
Cc026552	448	379						
Cc026553	322	120	XP_011001756.1					
Cc016559	114	140	XP_014524583.1	*Vigna radiata*	Fabaceae	Fabales	Albumin*[8]	Defence
Cc000814	109	124	KRH20687.1	*Glycine max*	Faboidae		Albumin*[8]	Defence
Cc000809	126	129	CAH05245.1	*Philenoptera violaceae*			Albumin*[8]	Defence
Cc000810	111	121						
Cc016556	126	130						
Cc016557	130	129						
Cc016558	128	134						
Cc026087	354	219	XP_014519530.1	*Vigna radiata*			F-box protein	unknown
Cc013727	481	352	XP_015941539.1	*Arachis duranensis*			F-box protein	unknown
Cc023681	542	359	KYP33191.1	*Cajanus cajan*			FAR1-related seq. 5	Development
Cc027215	797	1024	XP_006597152.1	*Glycine max*			CES101 G-type lectin receptor-like kinase	Defence
Cc029557	496	526	XP_003540336.1	*Glycine max*			Anthocyanin malonyl-transferase, BAHD-type	Secondary metabolism
Cc037361	431	440						
Cc038456	496	695	XP_013469345.1	*Medicago truncatula*			Clan71/CYP76-type cytochrome P450 monooxygenase	unknown
Cc038461	496	695						
Cc002675	215	325	XP_016181845.1	*Arachis ipaensis*	Arachis		Ankyrin repeat	unknown
Cc030668	213	305						
Cc025264	225	207	XP_015965546.1	*Arachis duranensis*	Arachis		Replication factor-A CTD-like	unknown
Cc026335	273	187						
Cc008645	277	171	KYP69131	*Cajanus cajan*	Cajanus		Hypothetical protein	unknown
Cc017139	400	434	XP_010091000.1	*Morus notablis*	Morus	Rosales	F-box protein	unknown
Cc025269	393	408						
Cc035460	288	305						
Cc041790	401	430						
Cc007716	479	543	XP_015900204.1	*Ziziphus jujuba*	Rosales		Receptor kinase	unknown
Cc010321	471	528						
Cc012785	225	285	XP_015901651.1	*Ziziphus jujuba*	Ziziphus		23 kDa jasmonate-induced protein-like	unknown
Cc029710	309	214	XP_009364679.1	*Pyrus x bretschneideri*	Pyrus		Zinc finger BED domain containing protein	Transcription
Cc040333	861	204						
Cc002477	329	586	XP_018466532.1	*Raphanus sativus*	Brassicaceae	Brassicales	Strictosidine synthase-like 1*[9]	Secondary metabolism
Cc014430	346	646						
Cc018763	484	472	XP_012488091.1	*Gossypium raimondii*	Gossypium	Malvales	F-box protein	unknown
Cc024284	379	355						
Cc018767	768	412	XP_016729221.1	*Gossypium hirsutum*	Malvaceae		Subtilisin-like endopeptidase	unknown
Cc006070	414	152	XP_017985368.1	*Theobroma cacao*	Theobroma		Uncharacterized protein	unknown

continued on next page

continued from previous page

Cuscuta protein		Best hit in NCBI nr			Taxonomic unit containing all hits within 40% relative cutoff		Predicted function or domain	MapMan category
**#**	**amino acids**	**Bit-score**	**NCBI Acc. #**	**Species**	**Taxon**	**Order**		
Cc015818	252	330	ABU87404.1	*Salvia miltiorrhiza*	Lamiales	Lamiales	lectin-like receptor kinase SMLII	Defence
Cc035515	436	631	XP_011099700.1	*Sesamum indicum*			Xyloglucan-specific endoglucanase inhibitor	Defence
Cc036869	692	638						
Cc017975	637	726	XP_012829248.1	*Erythranthe guttata*			Late blight resistance protein homolog RIA-3	Defence
Cc021598	635	153	KZN03945.1	*Daucus carota*	Daucus	Apiales	hypothetical protein	unkown
Cc021599	482	267	KZN03944.1	*Daucus carota*			Uncharacterized protein, Kelch motif	unknown
Cc005440	536	283						
Cc013112	482	260						
Cc021601	468	258						
Cc021517	287	206	XP_010689633.1	*Beta vulgaris*	Beta	Caryophyllales	Uncharacterized protein	unknown
Cc029100	308	298	XP_010676933.1	*Beta vulgaris*			Uncharacterized protein	unknown
Cc022090	460	184	XP_010682948.1	*Beta vulgaris*			F-Box/Kelch repeat	Defence
Cc022092	446	228						
Cc046675	447	221						
Cc002536	435	569	KMT04016	*Beta vulgaris*	Amarantha-ceae		Hypothetical protein	unknown
Cc018719	536	239	XP_010674821.1	*Beta vulgaris*			F-box protein CPR30-like	Defence
Cc018720	348	237						
Cc024237	364	236						
Cc024241	347	224						
Cc024246	365	229						
Cc038449	364	203						
Cc015713	355	220	XP_010672220.1	*Beta vulgaris*			F-box protein CPR30-like	Defence
Cc018710	544	236						
Cc018712	364	236						
Cc018723	375	219						
Cc021281	355	220						
Cc024247	406	183						
Cc021518	347	341	KNA19093.1	*Spinacia oleracea*	Spinacia		Hypothetical protein	unknown
Cc021523	345	326						
Cc017365	208	155	ACN30700.1	*Zea mays*	Poaceae	Poales	Uncharacterized protein	unknown

* Proteins for which HGT occurrence in *Cuscuta* has been reported previously are highlighted in grey and referenced at the end.

Supplementary Table 5. Full names and bin category descriptions for lost genes shown in Figure 4a.

Gene name	Protein description	Category (MapMan bin)
SGAT	Serine-glyoxylate transaminase	amino acid metabolism
COG0212	5,10-methenyl-THF synthetase	C1-metabolism
MENG	2-phytyl-1,4-naphthoquinone methyltransferase	Co-factor and vitamine metabolism
NSP2	NSP2 component of nodulation initiation complex	development
SAG101	SAG101 regulator of plant immunity	development
PPDK	Pyruvate pyrophosphate dikinase	gluconeogenesis / glyoxylate cycle
ALDH	NADP-dependent GAP dehydrogenase	glycolysis
SOT	Brassinosteroid sulfotransferase	hormone metabolism
DES-1-like	Delta-4 sphingolipid desaturase	lipid metabolism
CMO-like	Choline monooxygenase	lipid metabolism
RAM2	RAM2 mycorrhiza-related G3P acyl transferase	lipid metabolism
STS	Stachyose synthase	minor CHO metabolism
SIP1	Raffinose synthase	minor CHO metabolism
NSH3	Nucleosid hydrolase	nucleotide metabolism
HCEF1	Plastid fructose 1,6 bisphosphatase	PS
CEPD	CEPD regulatory peptide of NRT2 nitrate transporter	redox
RAM1	RAM1 mycorrhiza-related transcription factor	RNA
NIN	NIN-type transcription factor	RNA
NSP1	NSP1 component of nodulation initiation complex	RNA
ERN1	Ethylene Response Factor Required for Nodulation1	RNA
NFP	NFR5 nodulation-related receptor kinase	signalling
DMI2	SymRK nodulation-related receptor kinase	signalling
DMI3	CCaMK component of CCaMK-IPD3 kinase	signalling
CLH1/COR1	CLH1 Chlorophyllase	stress
EDS5	Plastid salicylic acid transporter	stress
EDS1	EDS1 regulator of plant immunity	stress
NRT2	NRT2-type nitrate transporter	transport
NHD	NHD-type plastid proton/sodium antiporter	transport
PHT2	PHT2 inorganic phosphate transporter	transport
HKT1	HKT1 sodium/potassium transporter	transport
IRT1	IRT 1 iron transporter	transport
NCS1	PLUTO nucleobase cation transporter	transport
aa transporter	HAAAP-type amino acid transporter	transport
TDT	TDT-type vacuolar di-/tricarboxylate transporter	transport
NAR2	Nar2 nitrate uptake accessory component	transport
VAPYRIN	VAPYRIN ankyrin repeat RF-like protein	transport
LRGB	PLGG plastid glycolate-glycerate transporter	not assigned
SGRL	SGRL-type Mg-dechelatase	not assigned
RP1	Regulatory kinase of PPDK	not assigned
CLD1	CLD1 chlorophyll dephytylase	not assigned
IPD3	IPD3 component of CCaMK-IPD3 kinase	not assigned
CERBERUS	CERBERUS ubiquitin ligase	not assigned

In addition, five Supplementary Data Files contain data that is too large to be displayed on a single page:

Supplementary Data 1: Orthogroups comprising genes from *Amaranthus hypochondriacus*, *Arabidopsis thaliana*, *Daucus carota*, *Mimulus guttatus*, *Oryza sativa*, *Sorghum bicolor*, *Solanum lycopersicum*, *Vitis vinifera*, *Populus trichocarpa*, *Dioscorea rotundata* and *Ipomoea nil*.

Supplementary Data 2: Expression data of HGT candidates.

Supplementary Data 3: Extrinsic information configuration file for AUGUSTUS.

Supplementary Data 4: List of species used for gene loss evaluation.

Supplementary Data 5: HGT candidate gene trees.

SUPPLEMENTARY REFERENCES

1. Huson, D. H. et al. MEGAN community edition - interactive exploration and analysis of large-scale microbiome sequencing data. *PLoS Comput. Biol.* **12**, e1004957 (2016).
2. Mirarab, S. & Warnow, T. ASTRAL-II: coalescent-based species tree estimation with many hundreds of taxa and thousands of genes. *Bioinformatics* **31**, i44-i52 (2015).
3. Stolzer, M. et al. Inferring duplications, losses, transfers and incomplete lineage sorting with nonbinary species trees. . *Bioinformatics* **28**, i409-i415 (2012).
4. Price, M. N., Dehal, P. S. & Arkin, A. P. FastTree 2--approximately maximum-likelihood trees for large alignments. *PLoS One* **5**, e9490 (2010).
5. Olsen, S. et al. Getting ready for host invasion: elevated expression and action of xyloglucan endotransglucosylases/hydrolases in developing haustoria of the holoparasitic angiosperm *Cuscuta*. *J. Exp. Bot.* **67**, 695-708 (2016).
6. Liu, C. et al. CpGAVAS, an integrated web server for the annotation, visualization, analysis, and GenBank submission of completely sequenced chloroplast genome sequences. *BMC Genomics* **13**, 715 (2012).
7. Simao, F. A., Waterhouse, R. M., Ioannidis, P., Kriventseva, E. V. & Zdobnov, E. M. BUSCO: assessing genome assembly and annotation completeness with single-copy orthologs. *Bioinformatics* **31**, 3210-3212 (2015).
8. Zhang, Y. et al. Evolution of a horizontally acquired legume gene, albumin 1, in the parasitic plant *Phelipanche aegyptiaca* and related species. *BMC Evol. Biol.* **13**, 48 (2013).
9. Zhang, D. et al. Root parasitic plant *Orobanche aegyptiaca* and shoot parasitic plant *Cuscuta australis* obtained Brassicaceae-specific strictosidine synthase-like genes by horizontal gene transfer. *BMC Plant Biol.* **14**, 19 (2014).
