# Supplementary material for: Footprints of parasitism in the genome of the parasitic flowering plant Cuscuta campestris
Source: Nat Commun. 2018 Jun 28;9:2515. doi: 10.1038/s41467-018-04344-z (PMC6023873; doi:10.1038/s41467-018-04344-z)
Supplement: Supplementary file 2 — Description of Additional Supplementary Files [file 41467_2018_4344_MOESM2_ESM.pdf]

Description of Additional Supplementary Files

File Name: Supplementary Data 1
Description: Orthogroups comprising genes from *Amaranthus hypochondriacus, Arabidopsis thaliana, Daucus carota, Mimulus guttatus, Oryza sativa, Sorghum bicolor, Solanum lycopersicum, Vitis vinifera, Populus trichocarpa, Dioscorea rotundata and Ipomoea nil.*

File Name: Supplementary Data 2
Description: Expression data of HGT candidates.

File Name: Supplementary Data 3
Description: Extrinsic information configuration file for AUGUSTUS.

File Name: Supplementary Data 4
Description: List of species used for gene loss evaluation.

File Name: Supplementary Data 5
Description: HGT candidate gene trees.
