# Supplementary material for: Footprints of parasitism in the genome of the parasitic flowering plant Cuscuta campestris
Source: Nat Commun. 2018 Jun 28;9:2515. doi: 10.1038/s41467-018-04344-z (PMC6023873; doi:10.1038/s41467-018-04344-z)

HGT candidate gene trees

For each novel *Cuscuta campestris* HGT candidate listed in Supplementary Table 4 that generated hits with more than one genus, a cladogram based on the alignments with an extended best hit sequence set as identified by MEGAN v.6.0.0 is shown. Partial sequence annotations were removed from the analysis. The trees were created with the multiple sequence alignment tool ClustalW2. Numbers at the nodes indicate bootstrap values from 1000 repetitions. *C. campestris* genes and their respective best hits as listed in Supplementary Table 4 are highlighted in blue.
The trees are listed in the same order as the genes are listed in Supplementary Table 4 with the respective putative donor gene given on top.

Putative donor sequences: XP_011001738.1 + XP_011001756.1

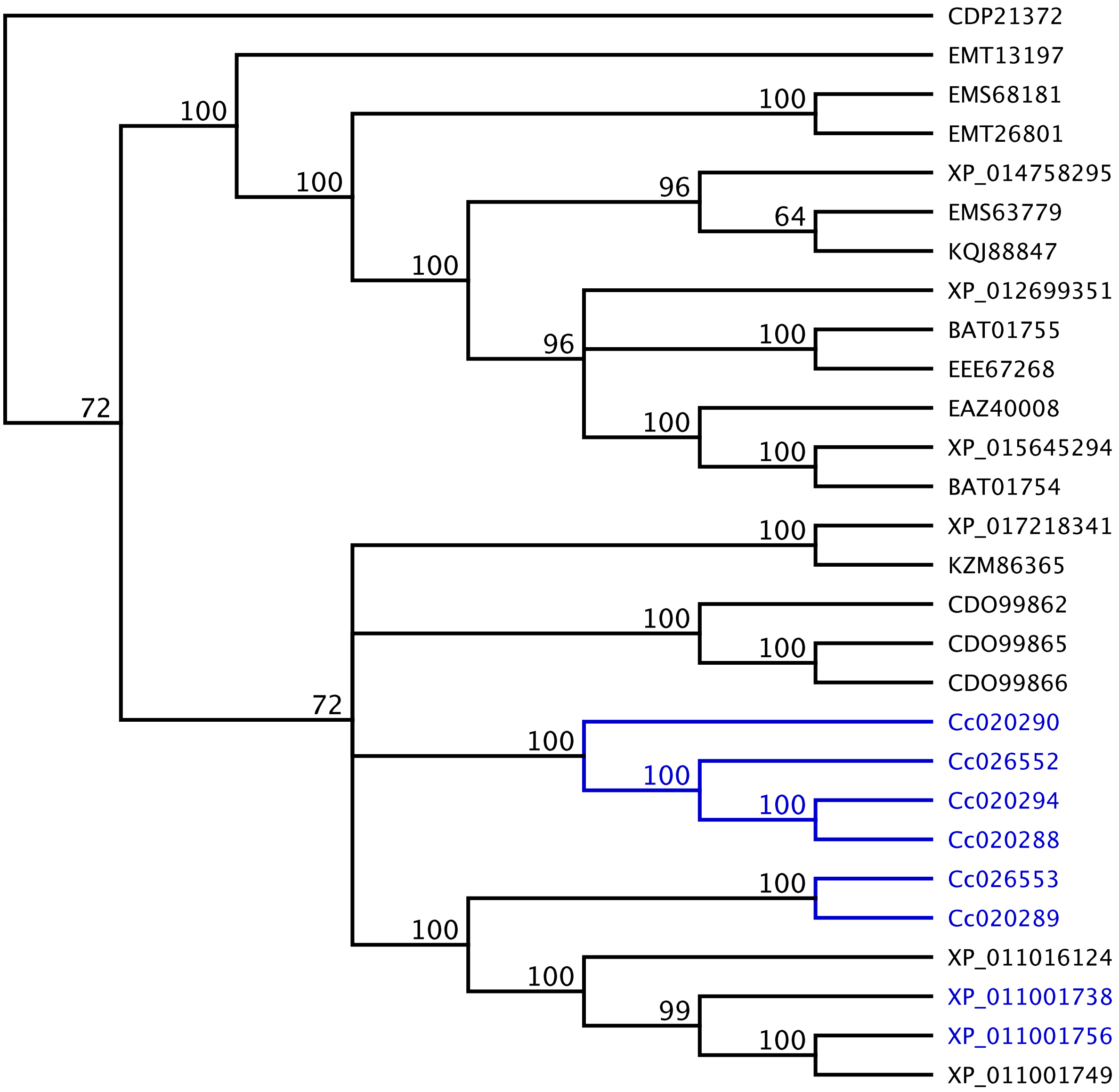

Putative donor sequences: XP_014519530.1 + XP_015941539.1

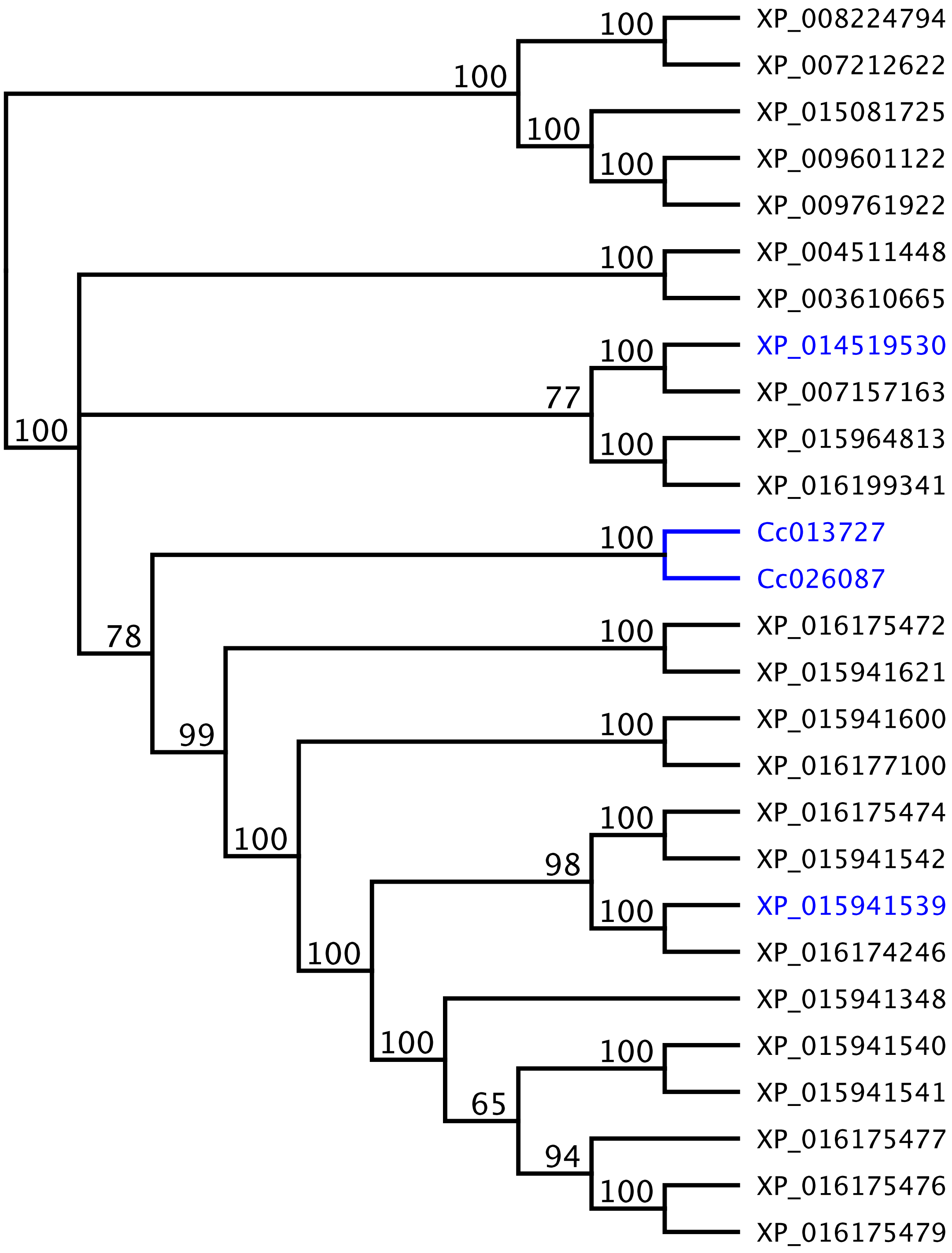

Putative donor sequences: KYP33191.1

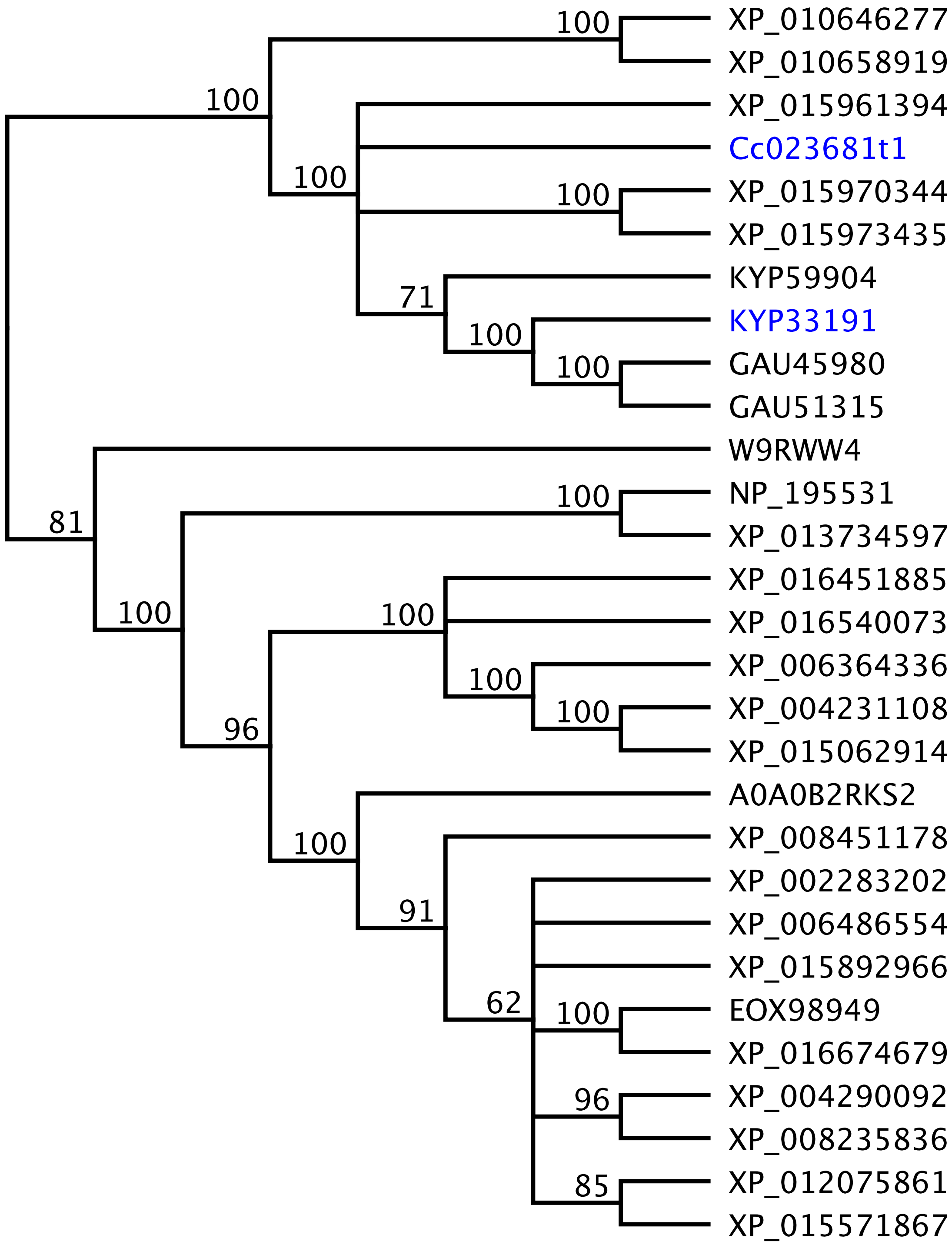

Putative donor sequences: XP_006597152.1

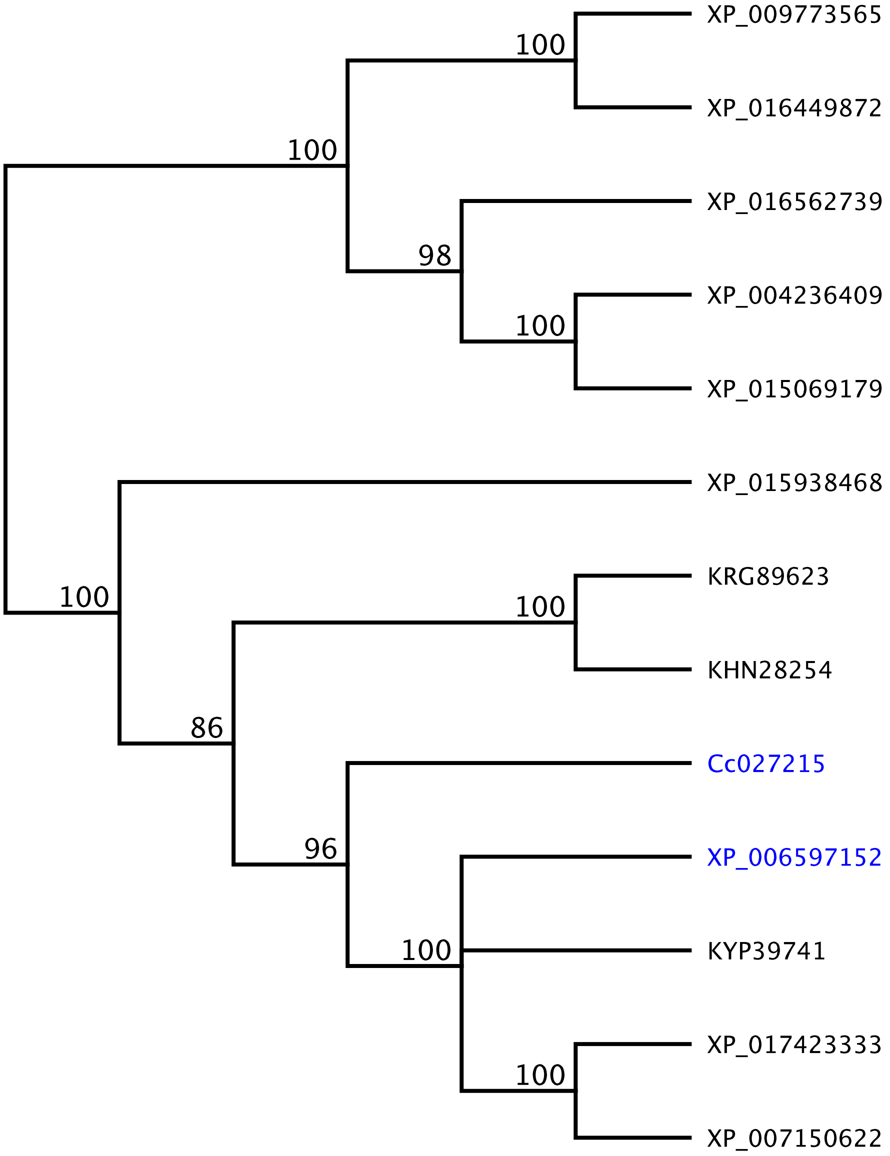

Putative donor sequences: XP_003540336.1

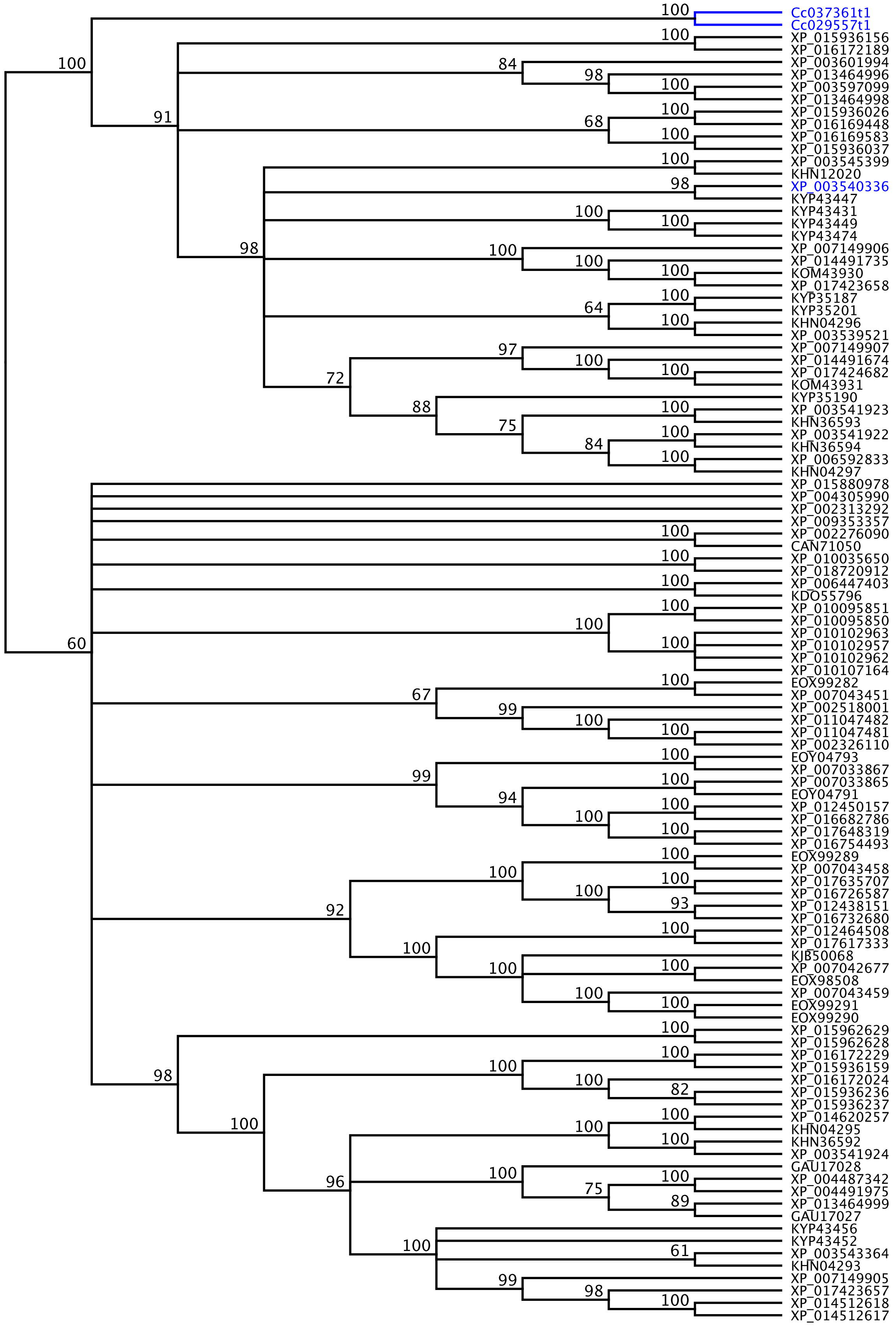

Putative donor sequences: XP_013469345.1

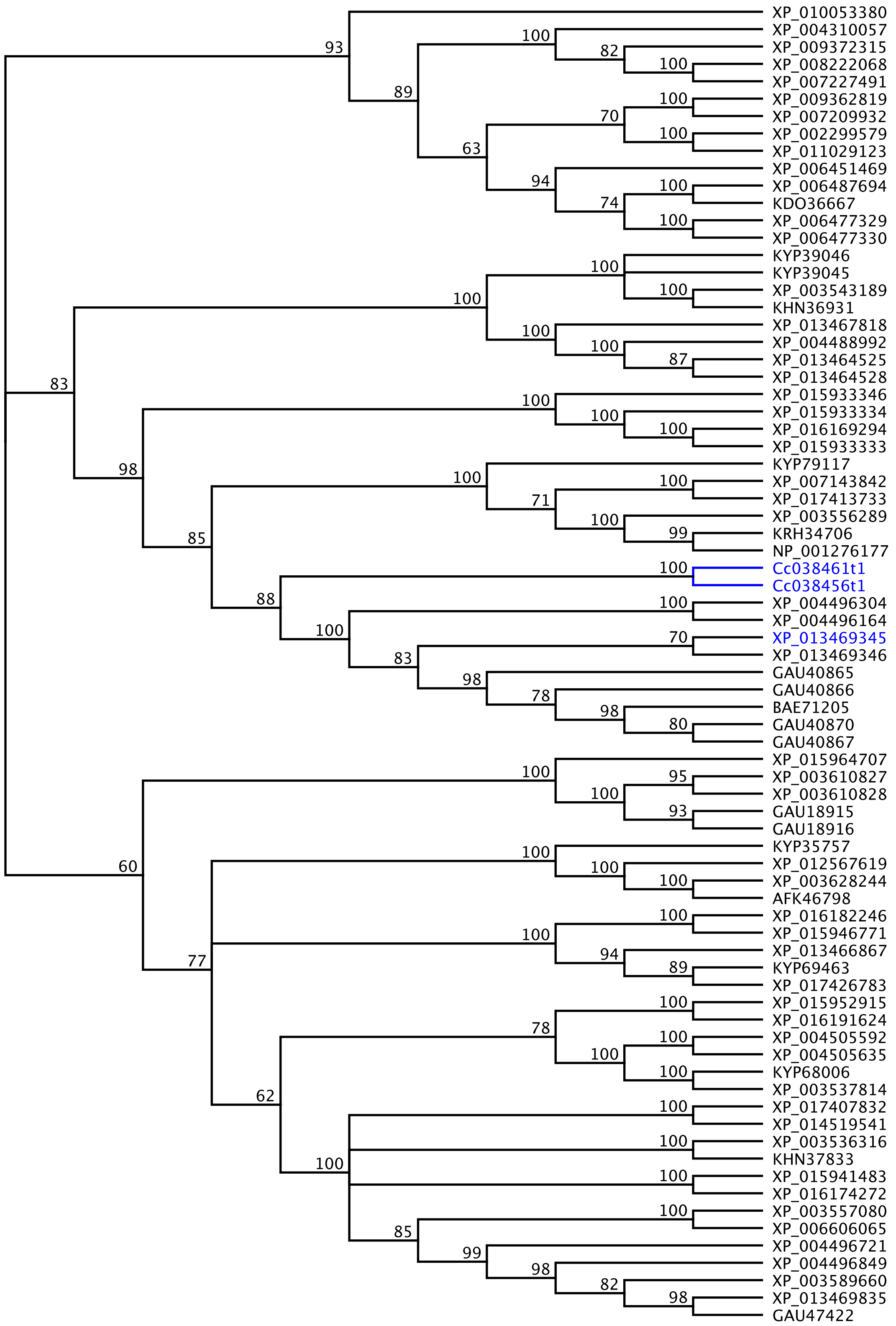

Putative donor sequences: XP_016181845.1

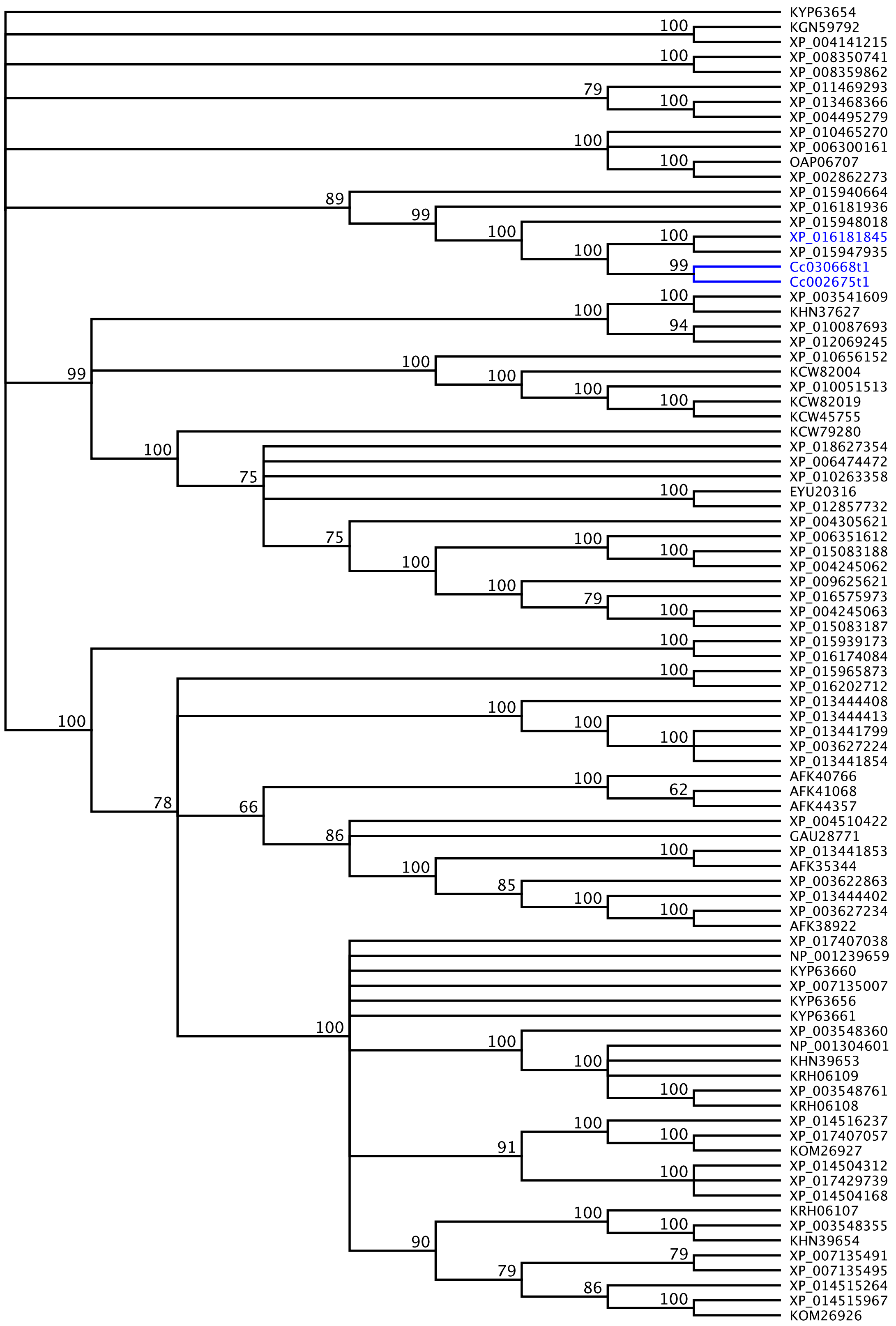

Putative donor sequences: XP_015965546.1

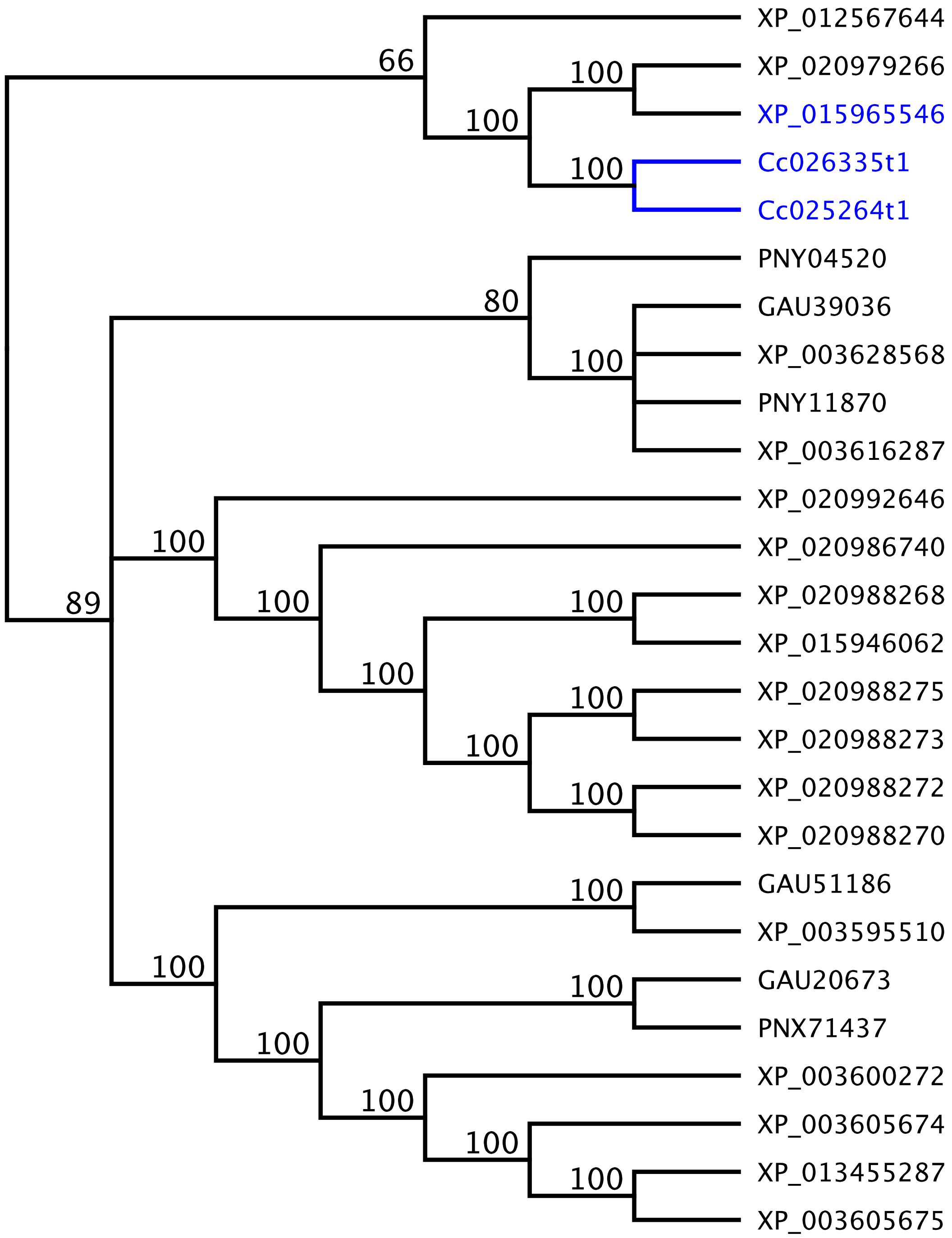

Putative donor sequences: XP_010091000.1

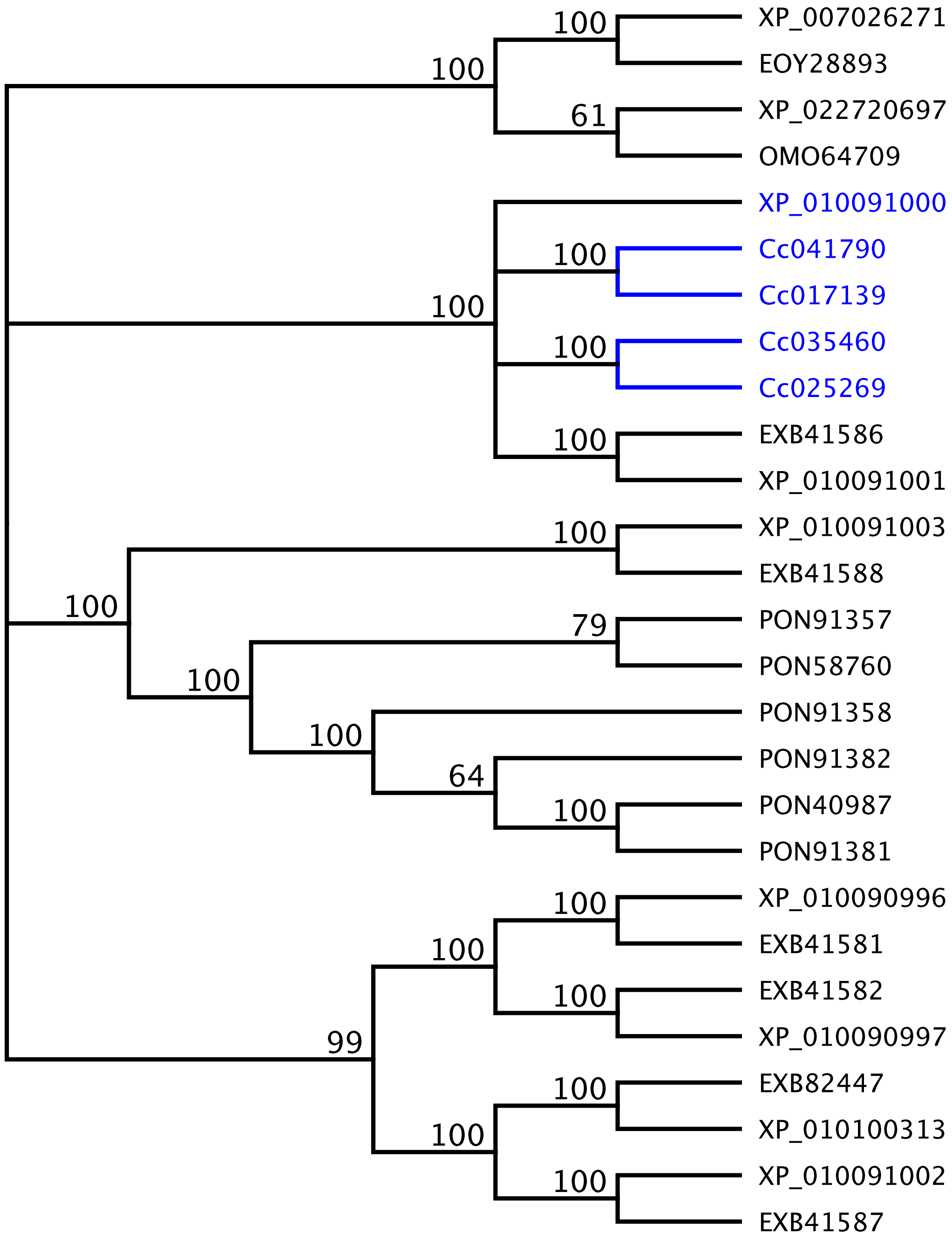

Putative donor sequences: XP_015900204.1

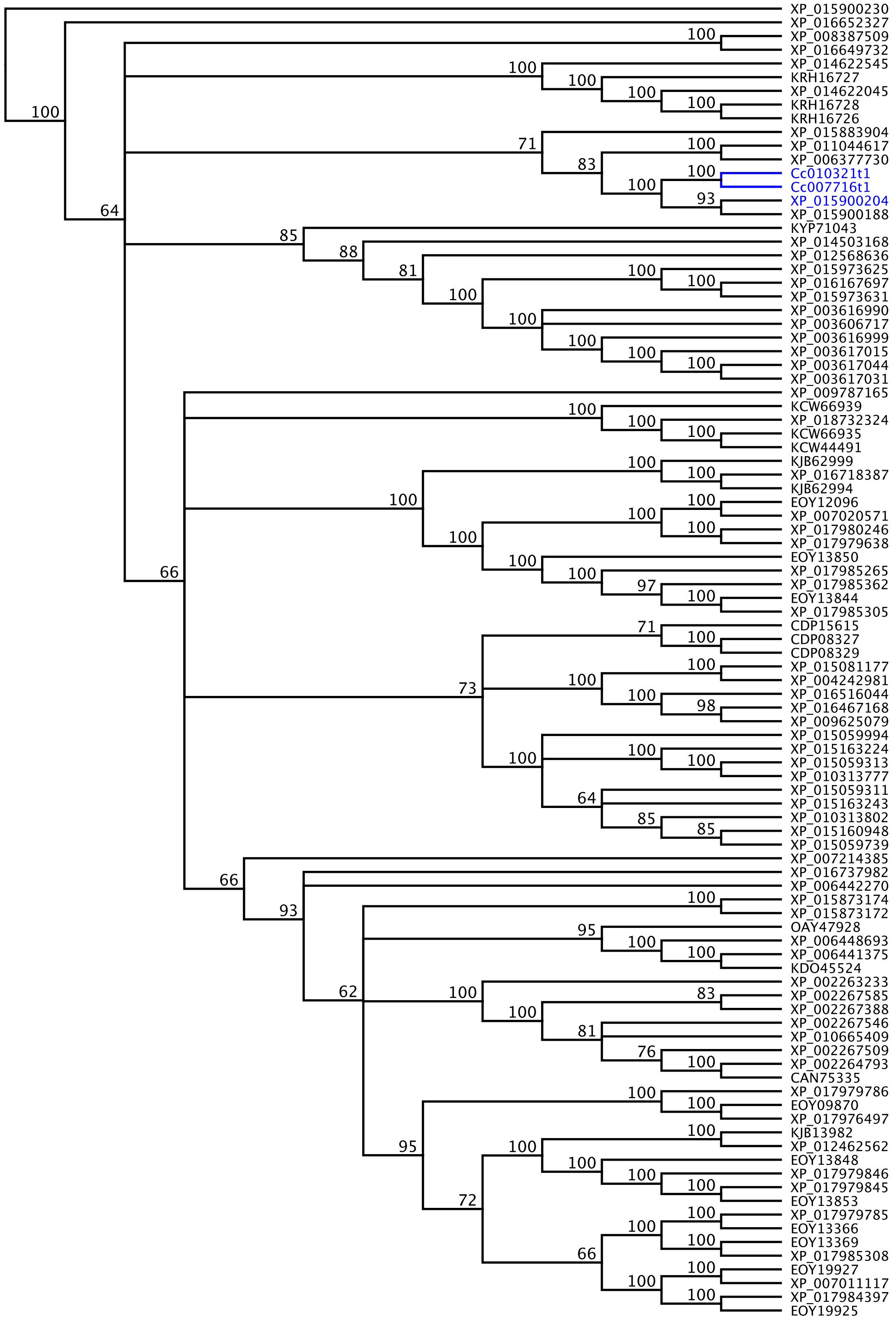

Putative donor sequences: XP_015901651.1

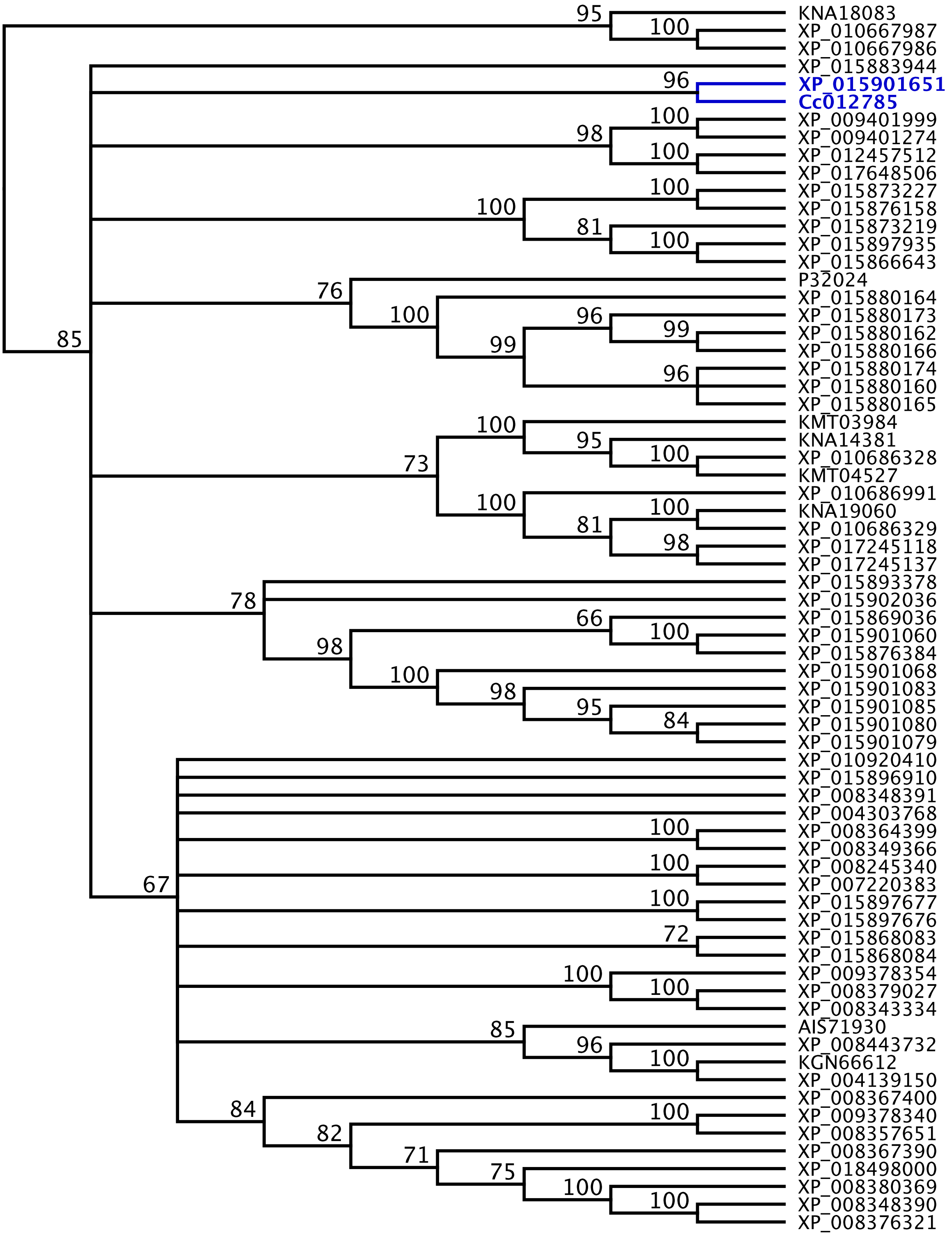

Putative donor sequences: XP_017178412 (original hit XP_009364679.1 has been removed from the NCBI database)

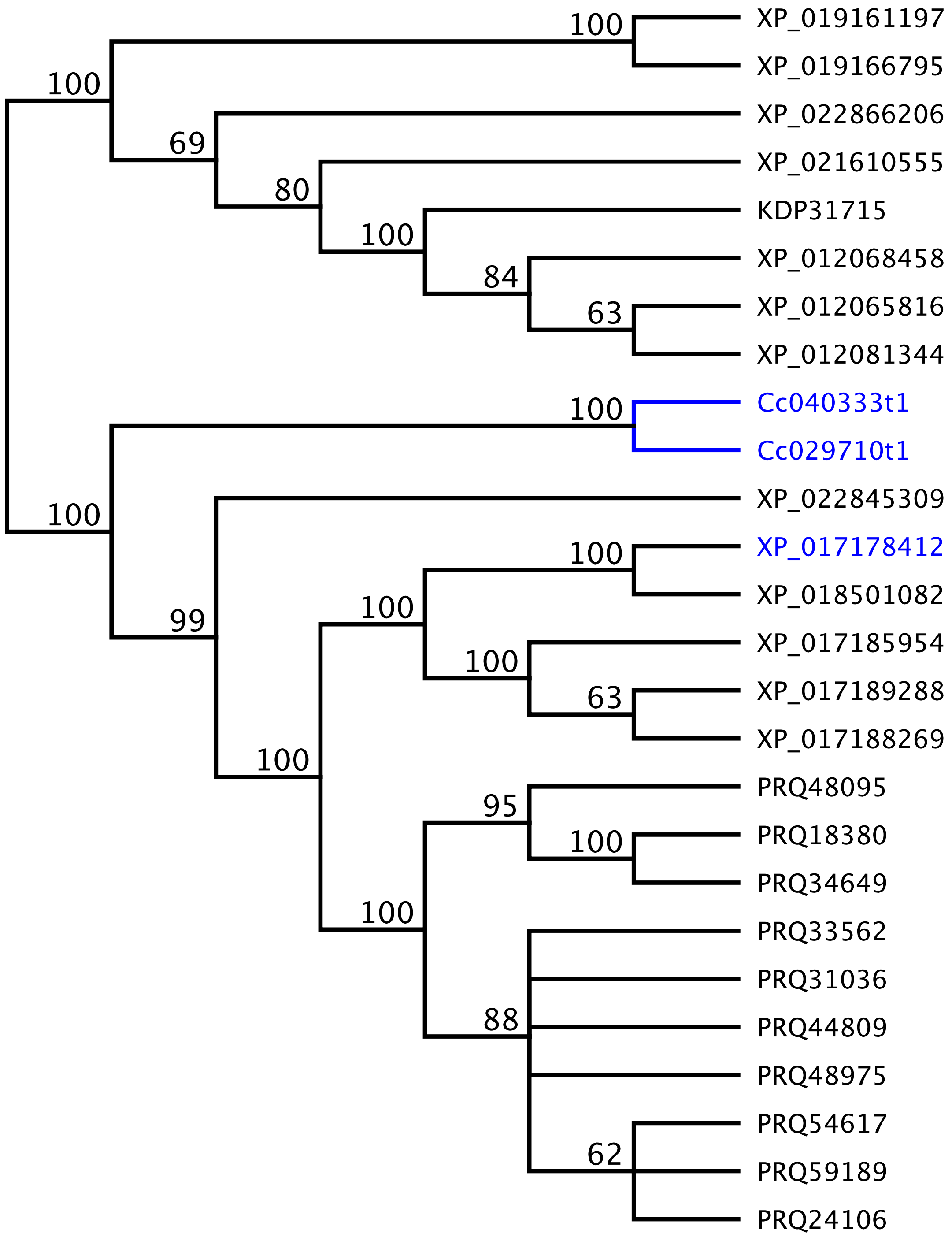

Putative donor sequences: XP_012488091.1

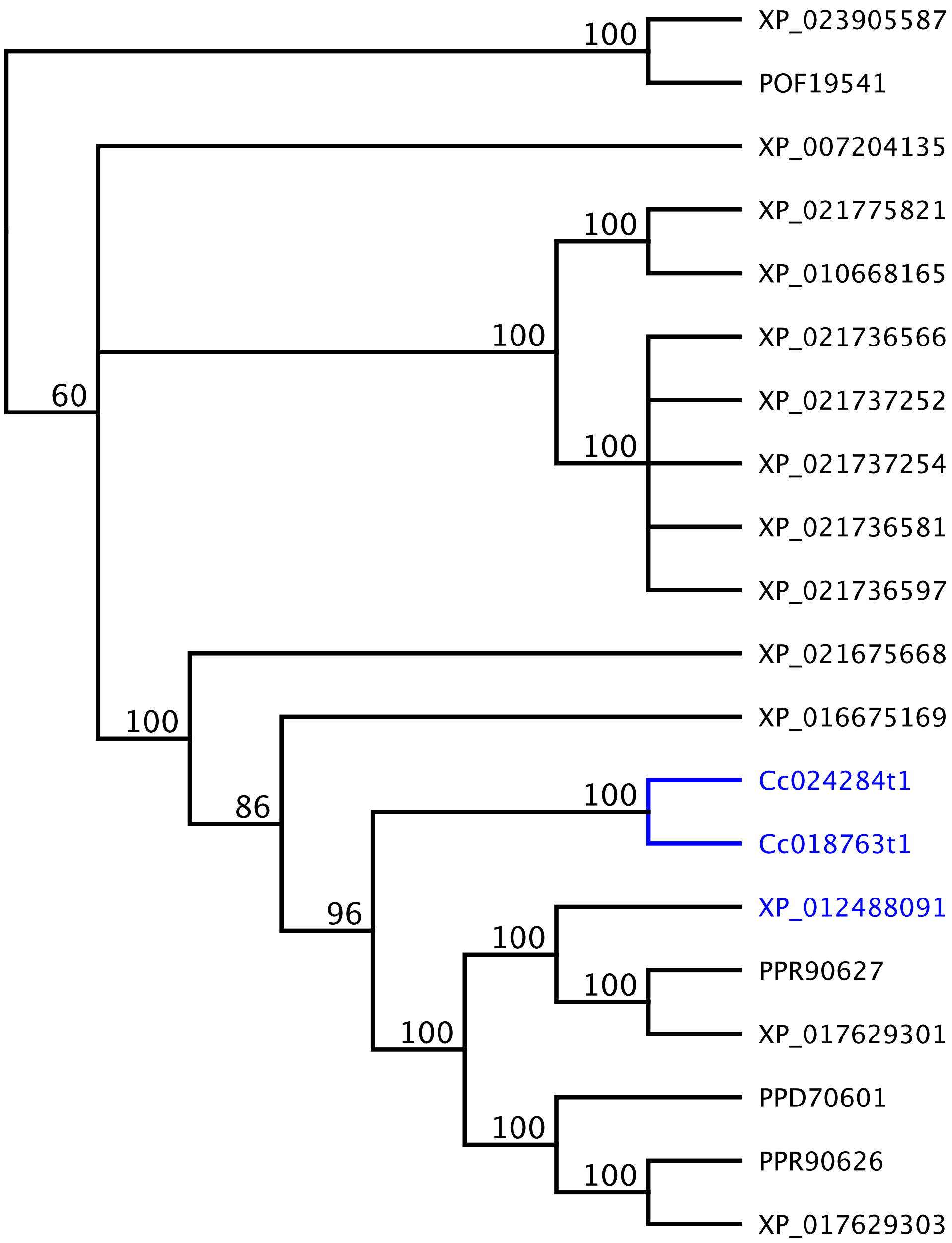

Putative donor sequences: XP_016729221.1

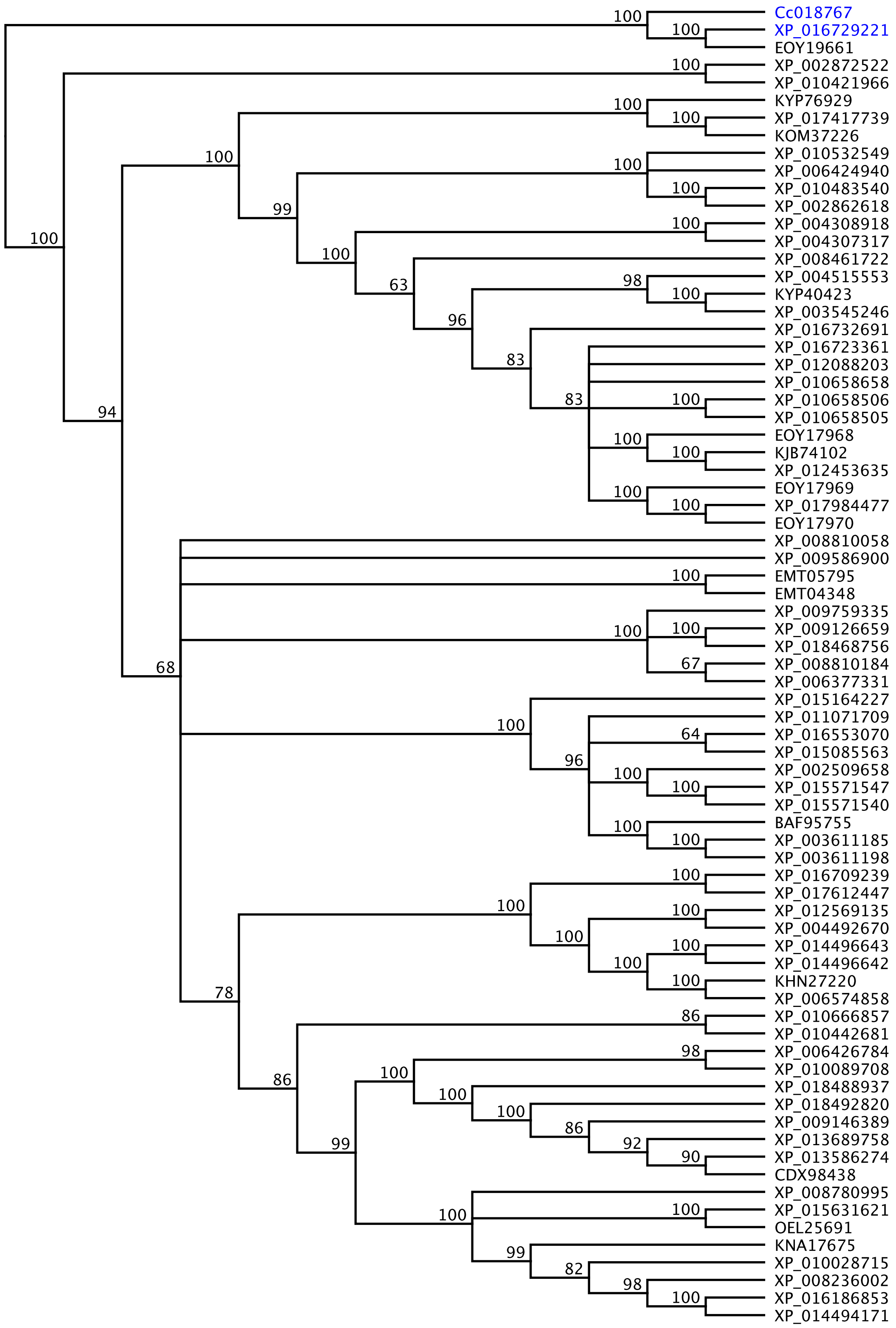

Putative donor sequences: ABU87404.1

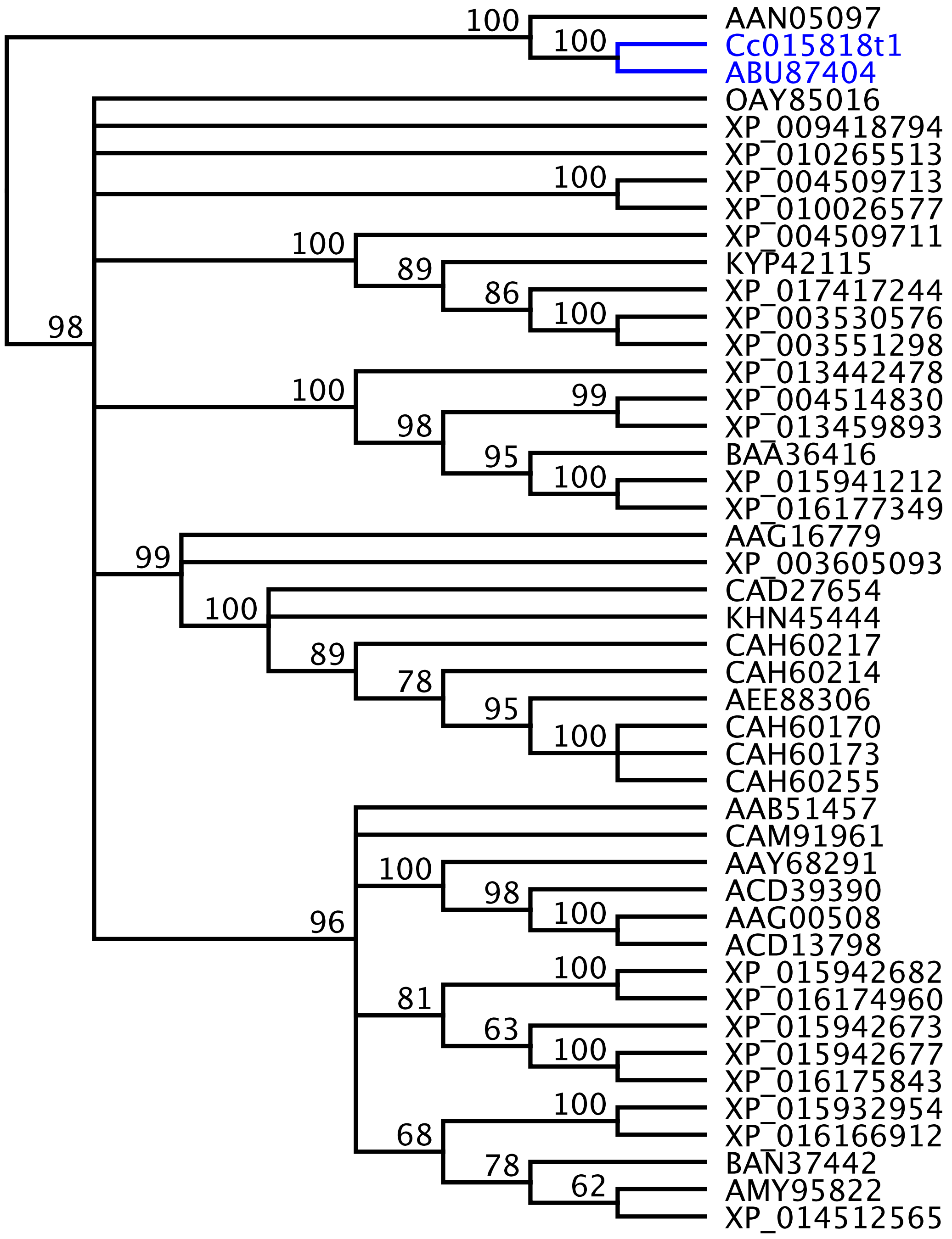

Putative donor sequences: XP_011099700.1

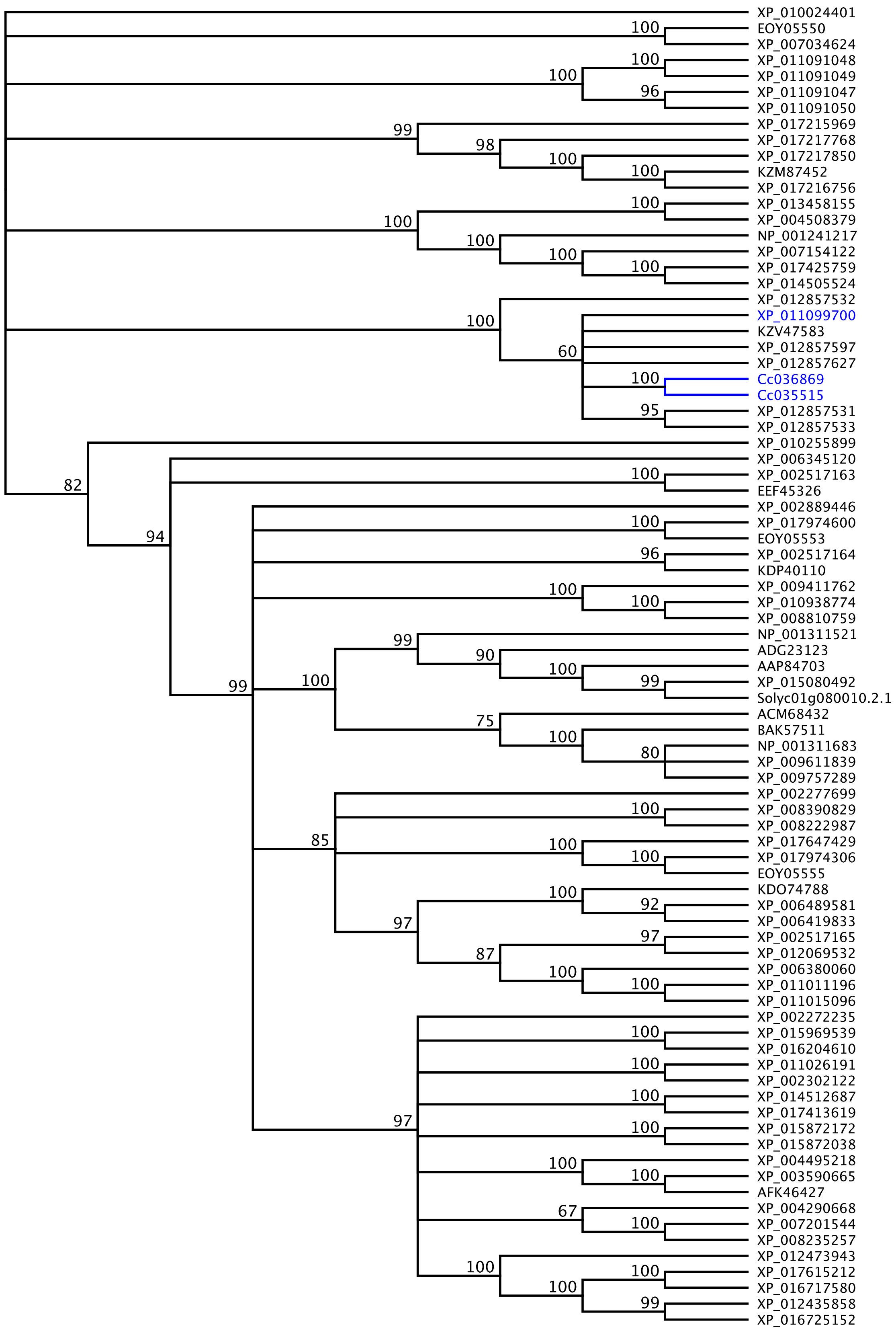

Putative donor sequences: XP_012829248.1

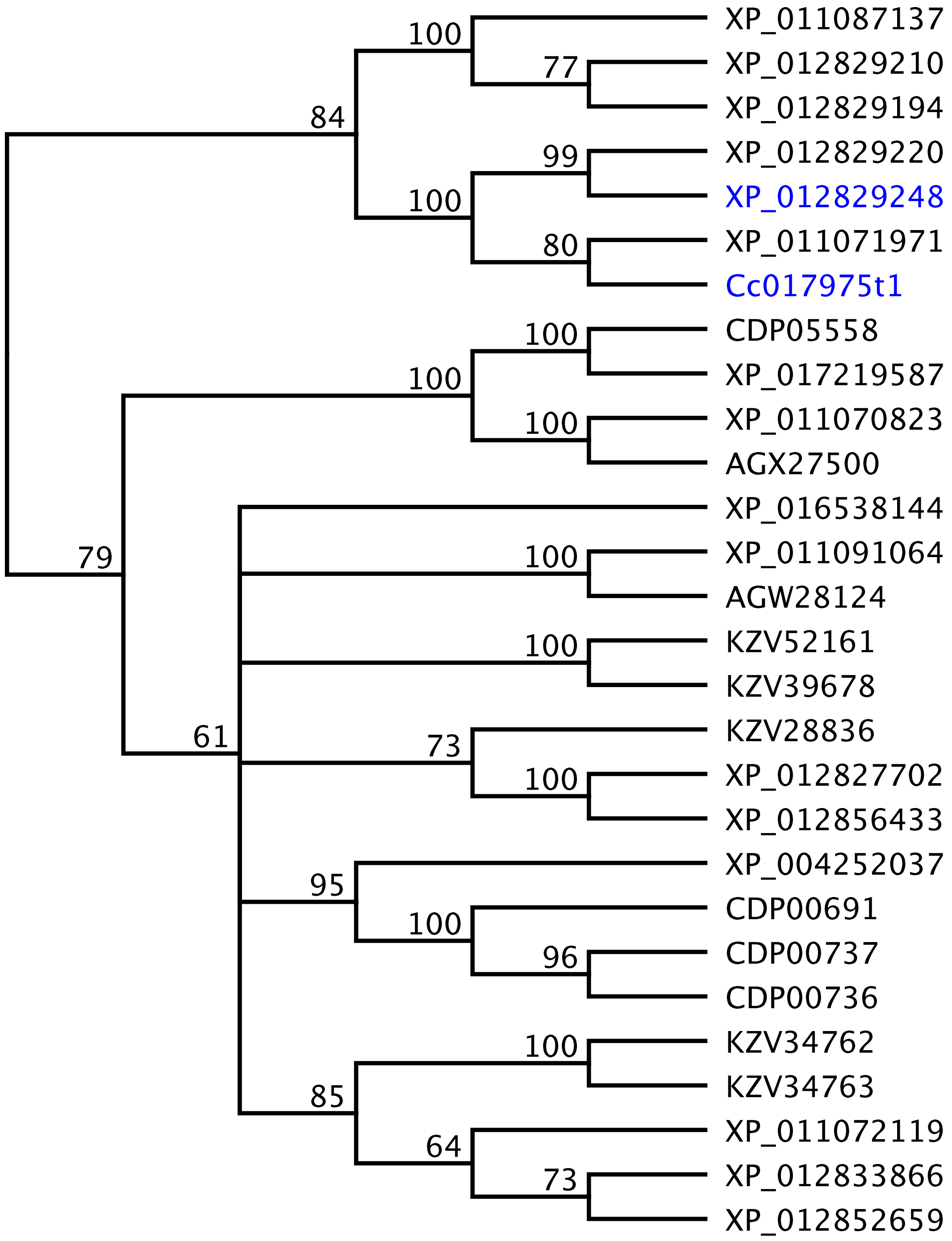

Putative donor sequences: XP_010676933.1

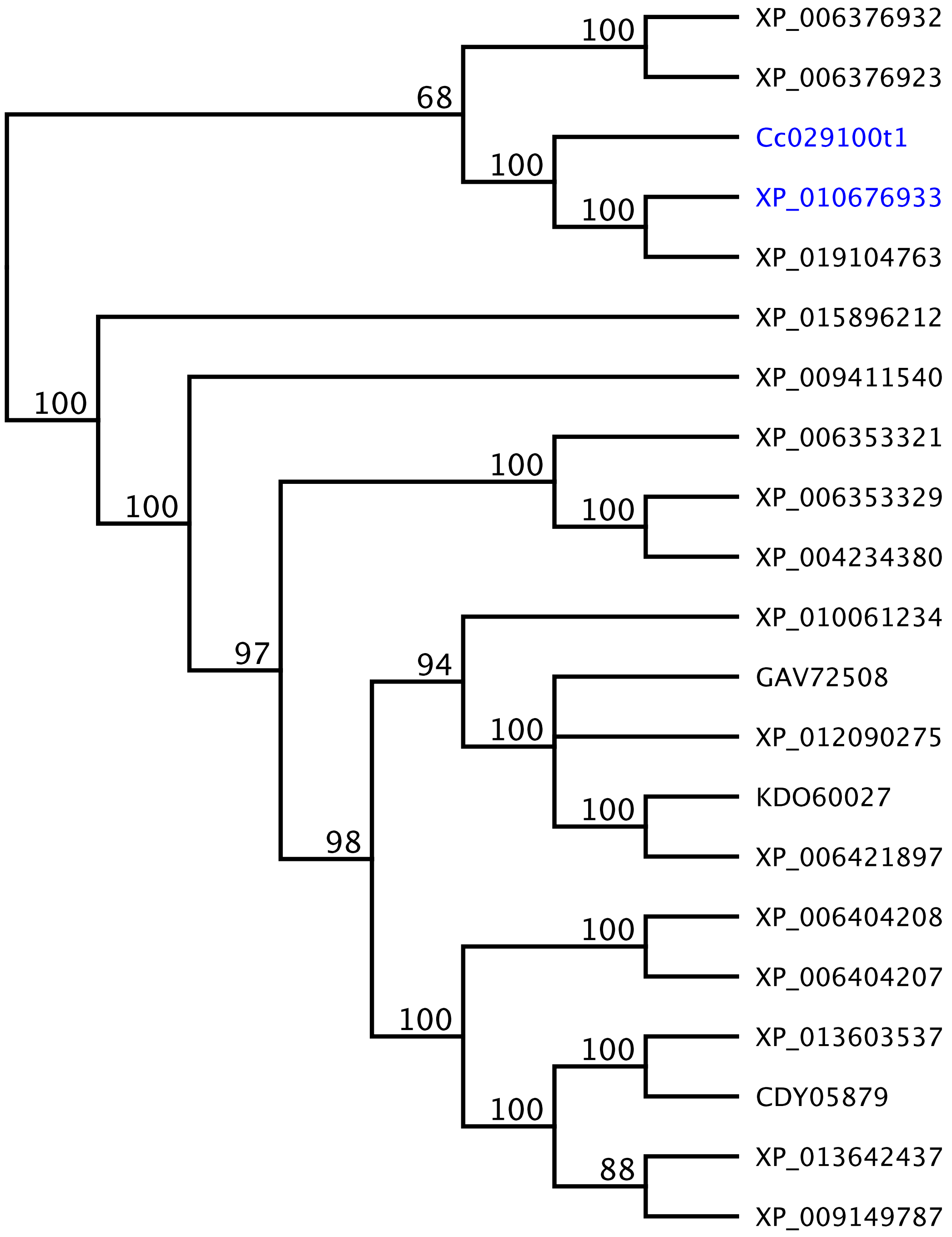

Putative donor sequences: XP_010682948.1

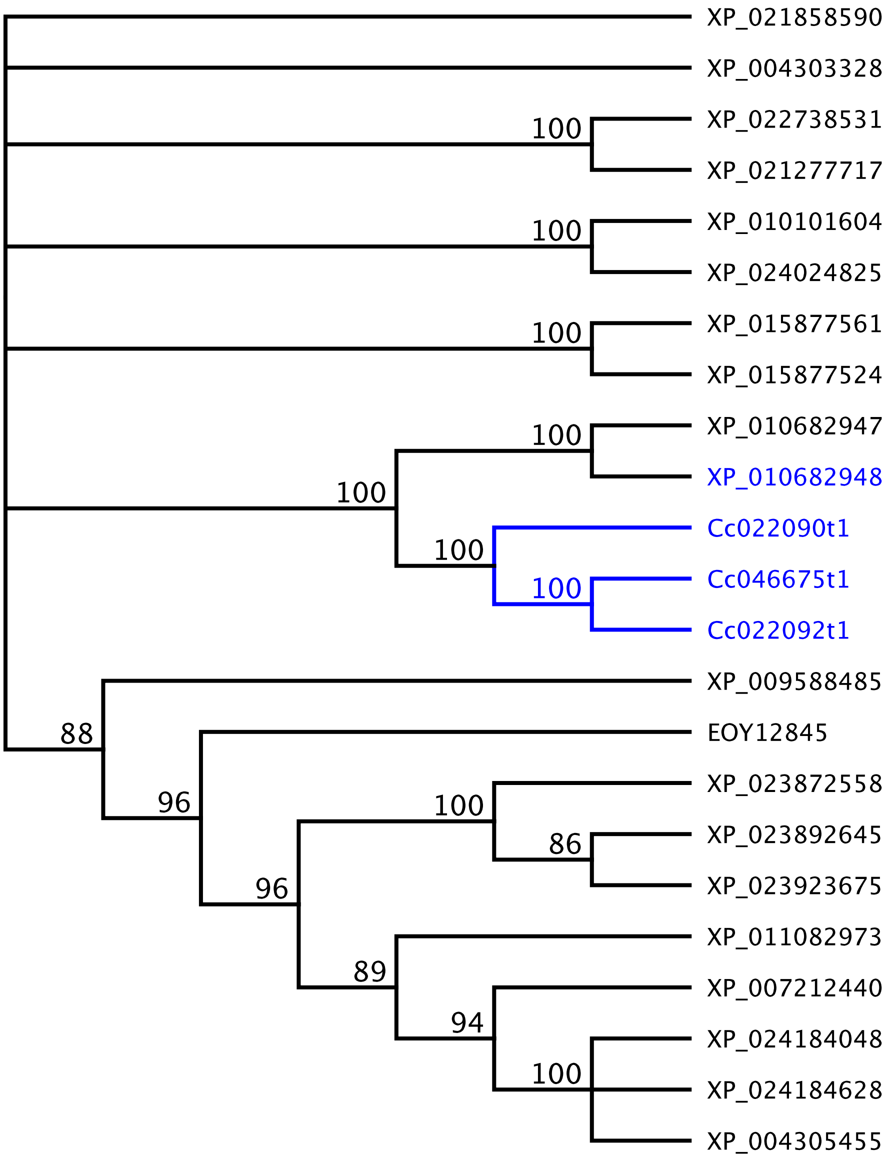

Putative donor sequences: KMT04016

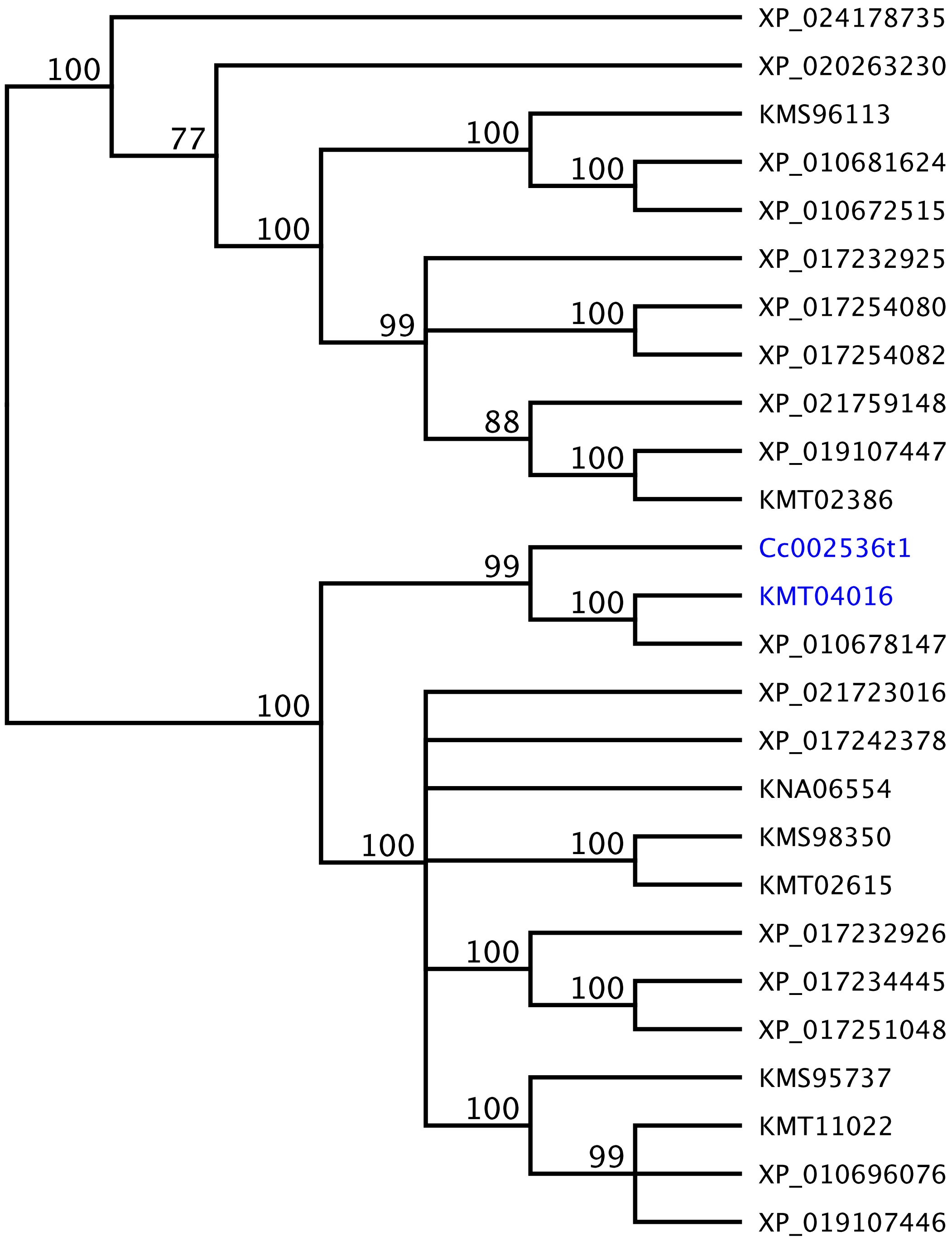

Putative donor sequences: XP_010674821.1

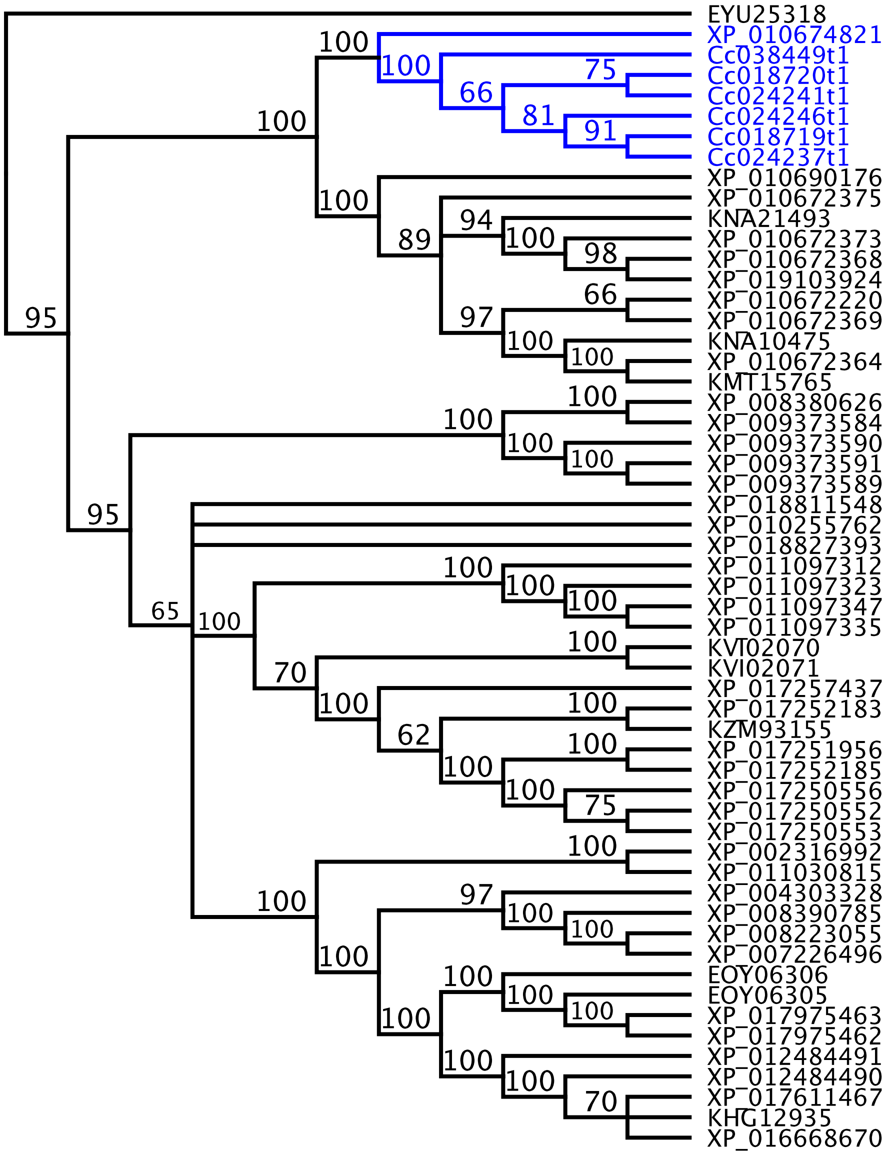

Putative donor sequences: XP_010672220.1

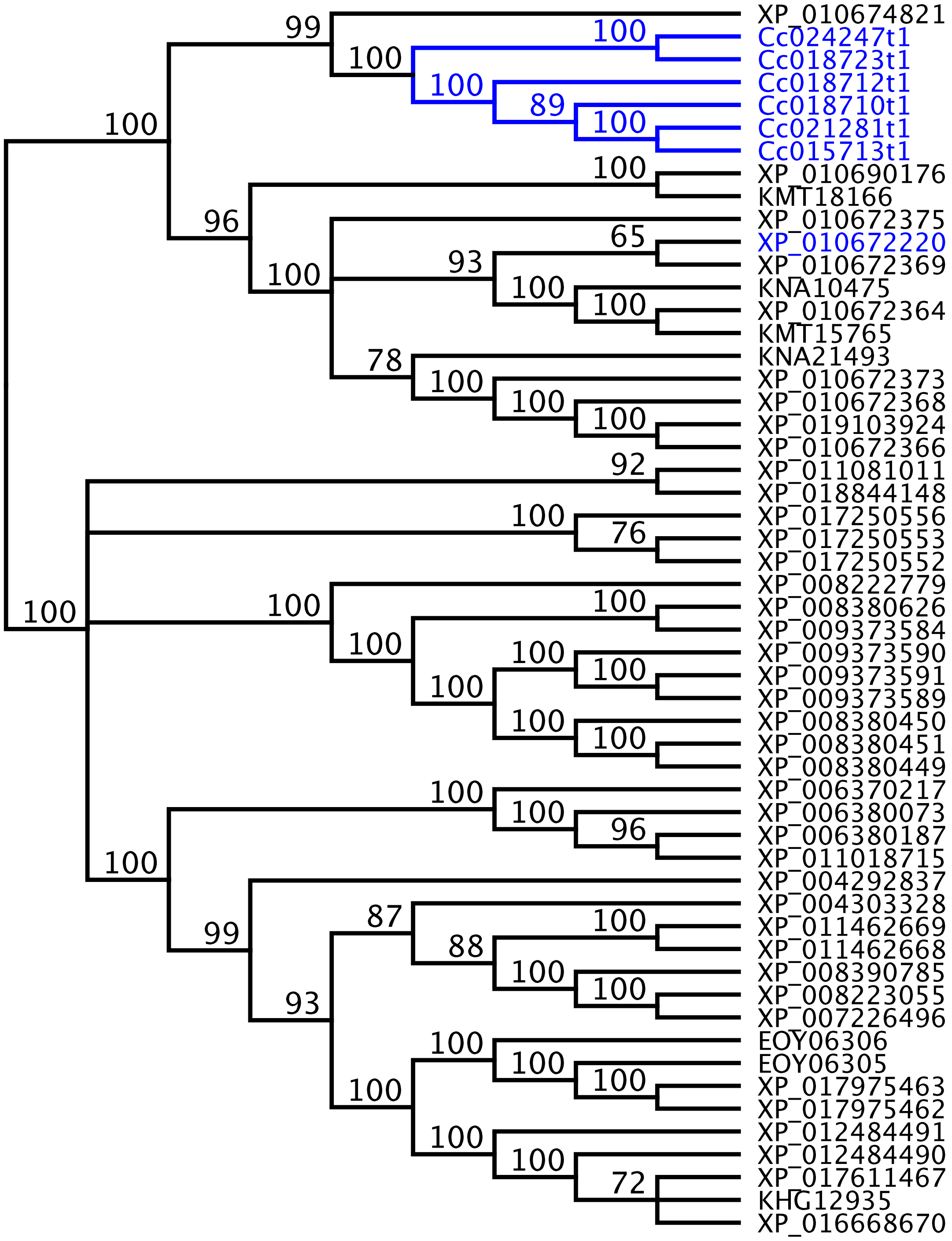

Putative donor sequences: KNA19093.1

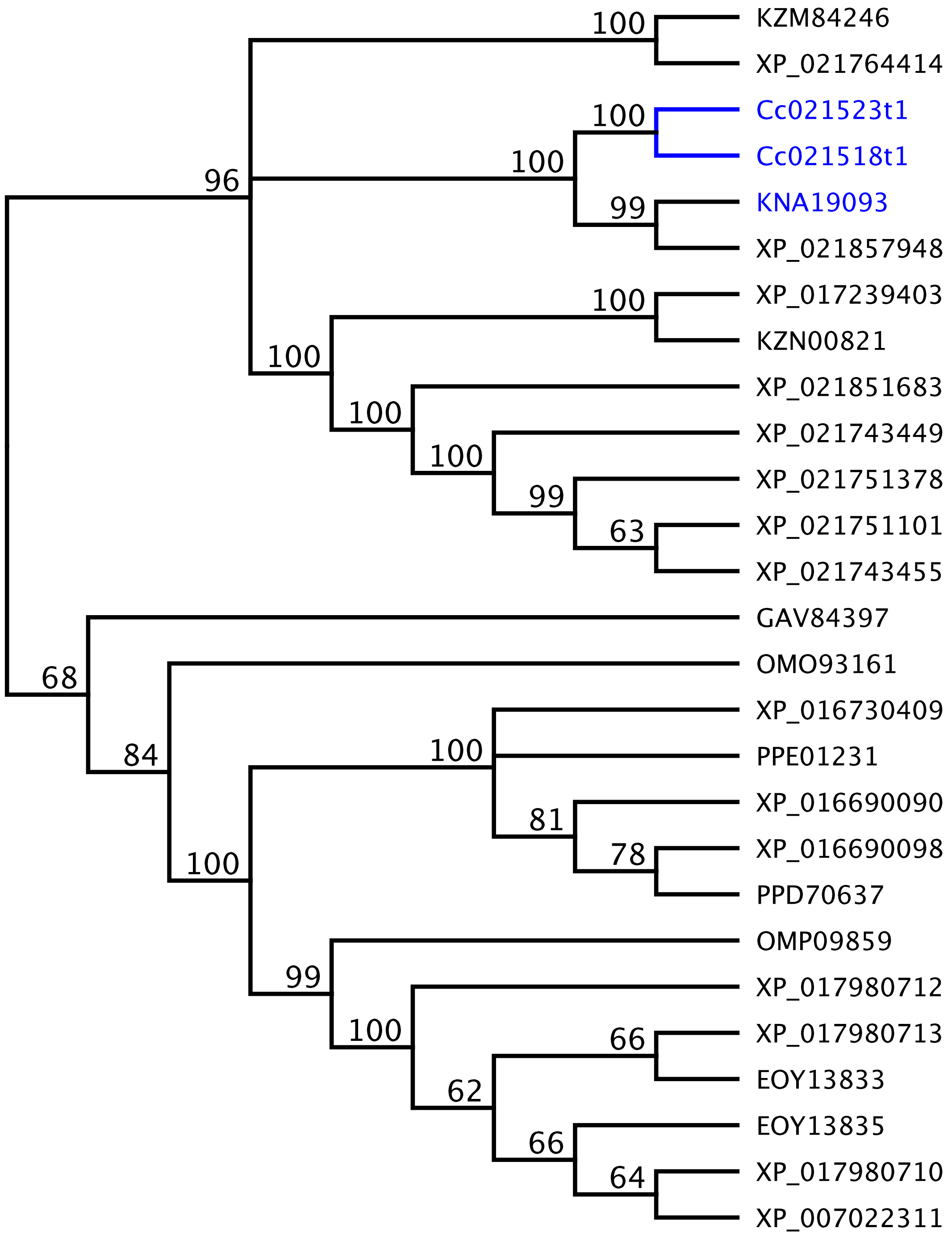

Putative donor sequences: ACN30700.1

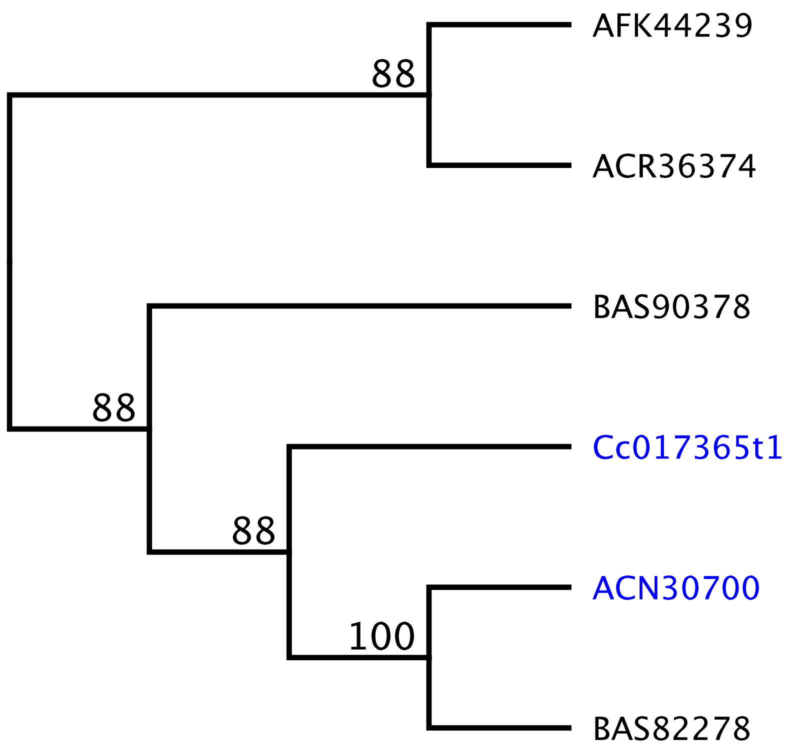

Supplement: Supplementary file 7 — Supplementary Data 5 [file 41467_2018_4344_MOESM7_ESM.pdf]
